# Supplementary material for: The extracellular contractile injection system is enriched in environmental microbes and associates with numerous toxins
Source: Nat Commun. 2021 Jun 18;12:3743. doi: 10.1038/s41467-021-23777-7 (PMC8213781; doi:10.1038/s41467-021-23777-7)
Supplement: Supplementary file 1 — Supplementary Information [file 41467_2021_23777_MOESM1_ESM.pdf]

Supplementary Information

The extracellular contractile injection system is enriched in environmental microbes and associates with numerous toxins

Alexander Martin Geller*, Inbal Pollin*, David Zlotkin, Aleks Danov, Nimrod Nachmias, William B Andreopoulos, Keren Shemesh, and Asaf Levy

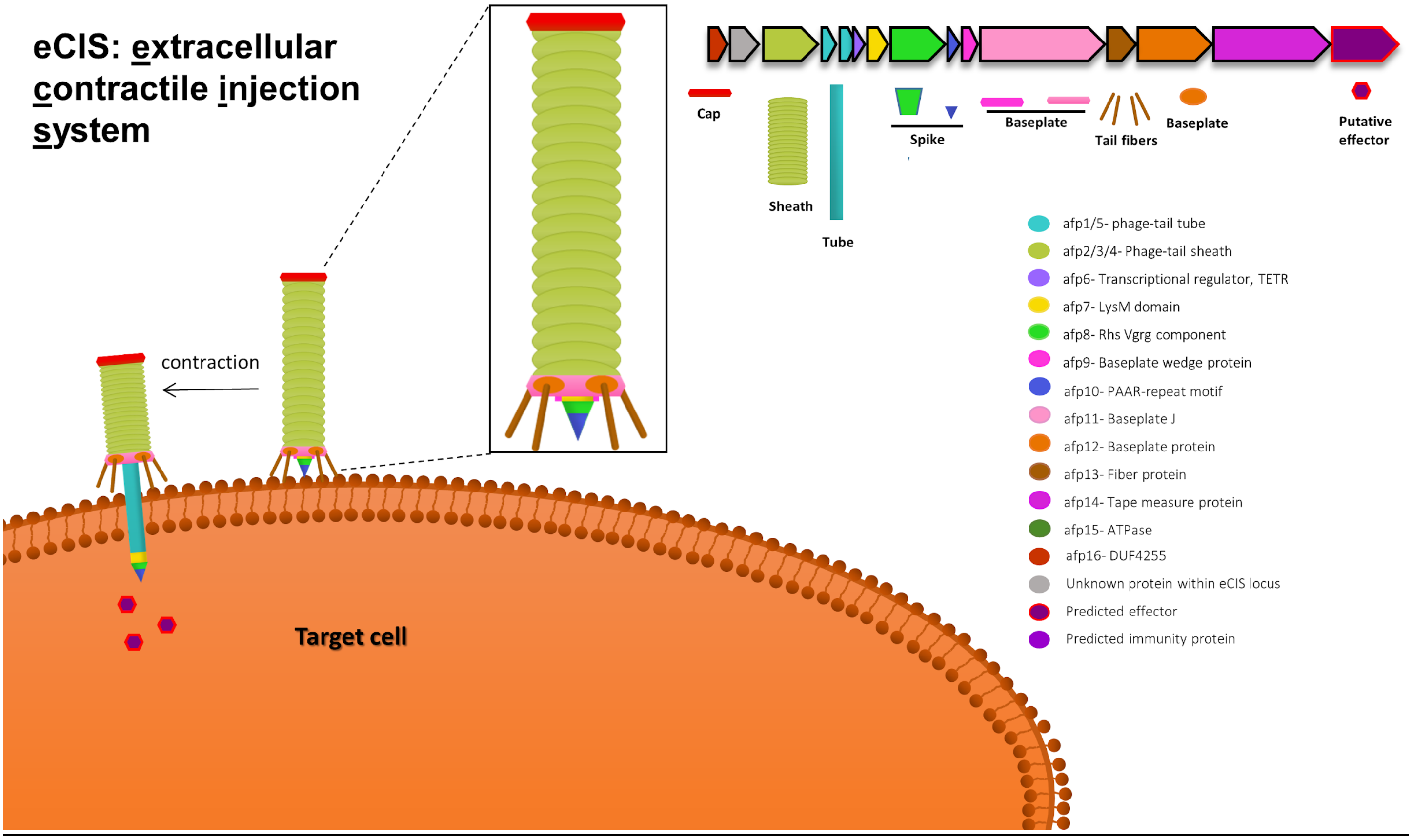

Supplementary Figure 1. eCIS structure. eCIS particles are encoded by an operon of 15-28 genes, a typical operon is shown, with cartoons showing corresponding structural components (right side). The core proteins encode a sheath that has a cap on one side and a baseplate on the other side. Inside the sheath there is a tube that ends with a sharp spike. The particle likely binds to receptors located on the target cell through tail fibers. Once the contact is established, the eCIS contracts, injects the tube, and eCIS-associated toxins (EATs) are released into the target cell (left side). Some of the toxins were reported to be located inside the hollow tube. The illustration is based on Jiang et al. [3]

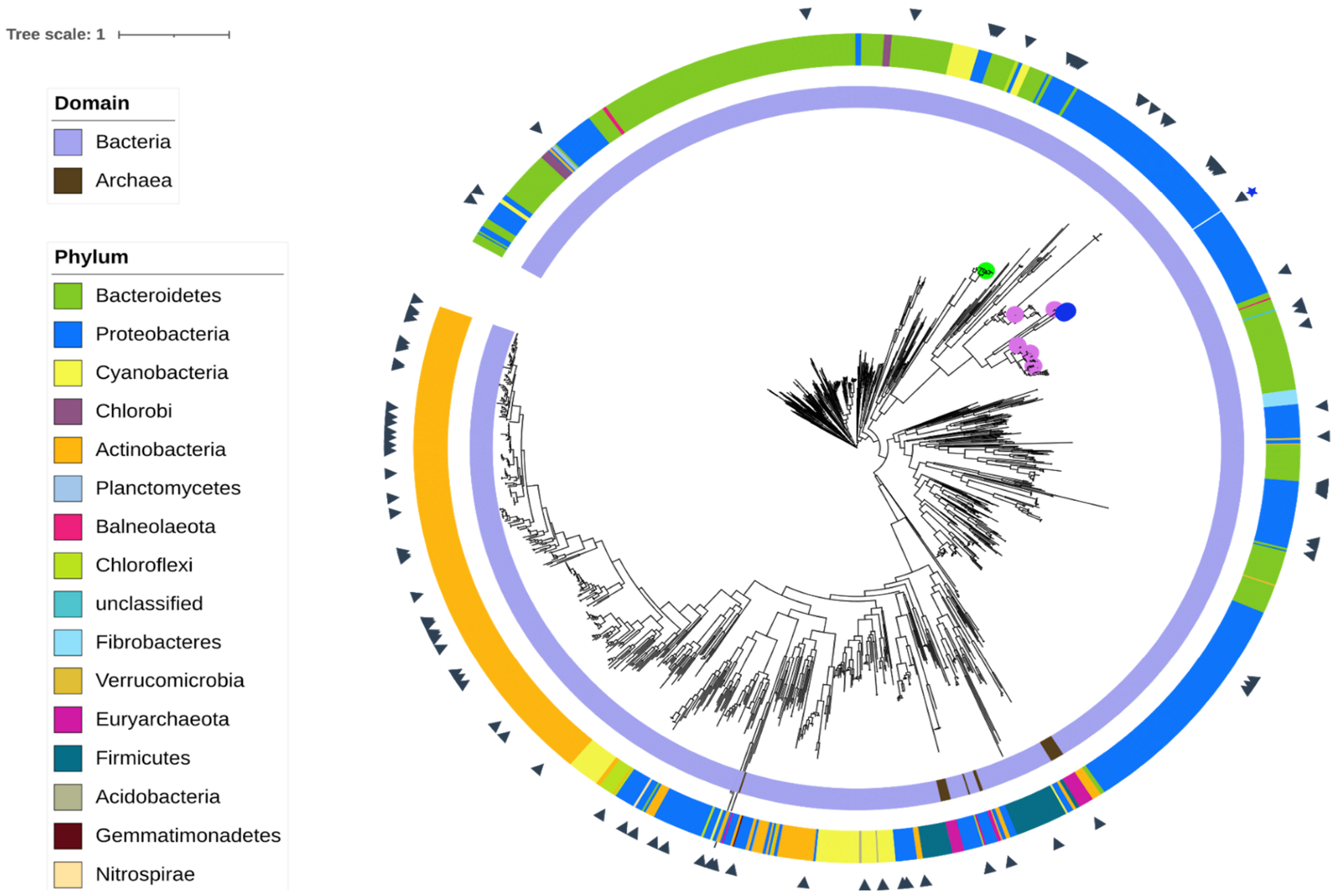

Supplementary Figure 2. Figure 1 zoomed in view. Inner ring represents the domain (legend on top left of figure). Outer ring represents Phylum (legend on left of figure). Triangles represent plasmid prediction by Deeplasmid (score >0.7); blue star (two o'clock) represents AFP, which is known to be encoded on a plasmid.

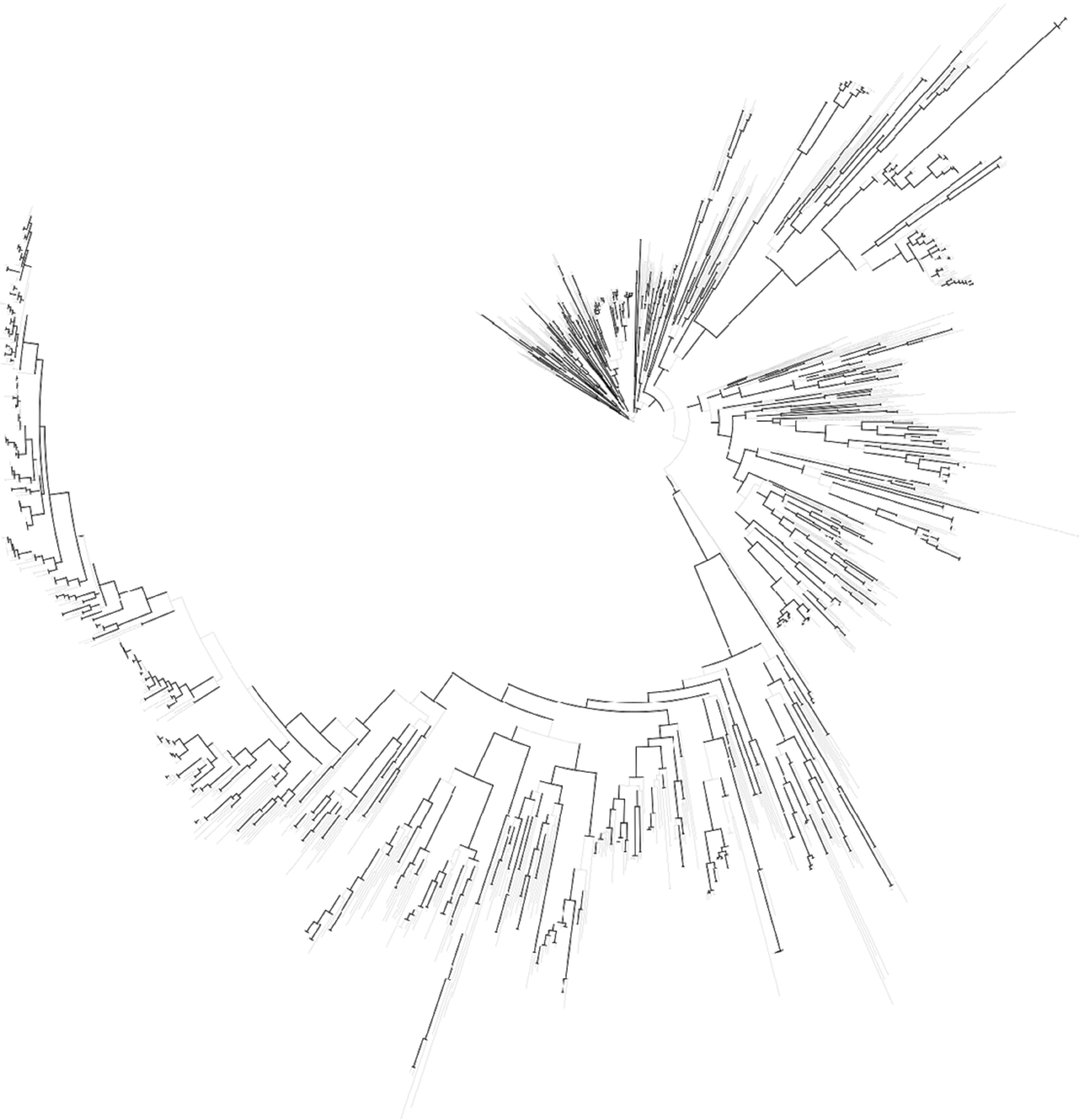

Supplementary Figure 3. Bootstrap values above 0.8 in eCIS tree. The phylogenetic tree of eCIS as shown in Figure 1 is re-plotted here with bold branches indicating bootstrap values that are greater than 0.8.

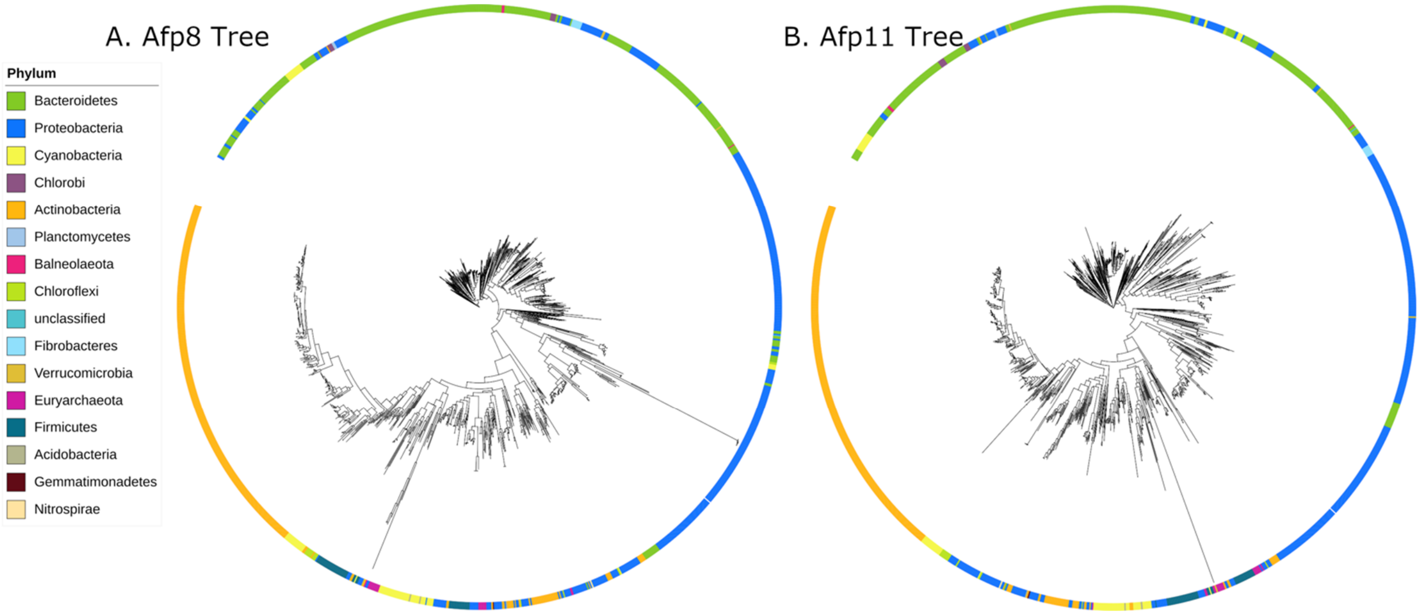

Supplementary Figure 4. Afp8 and Afp11 trees give similar results when plotted alone. Colors represent Phyla (legend in figure).

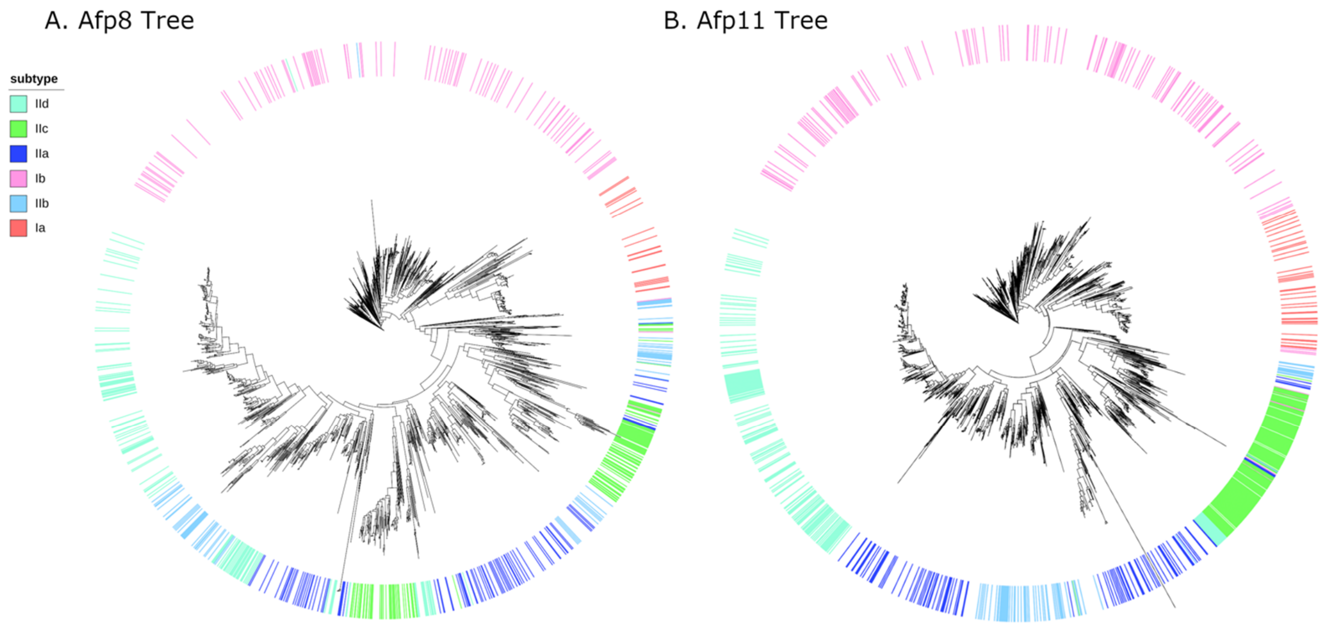

Supplementary Figure 5. Afp8 and Afp11 from dbeCIS and eCIStem. A large sample of Afp8 or Afp11 from dbeCIS[16] and the respective homologs from the current work were aligned and plotted with the same pipeline as for Figure 1. eCIS subtypes, as defined by dbeCIS, are marked in the ring surrounding the tree, with corresponding colors explained in the key. Those with "none" (white) are from eCIStem (Afp8 from this work).

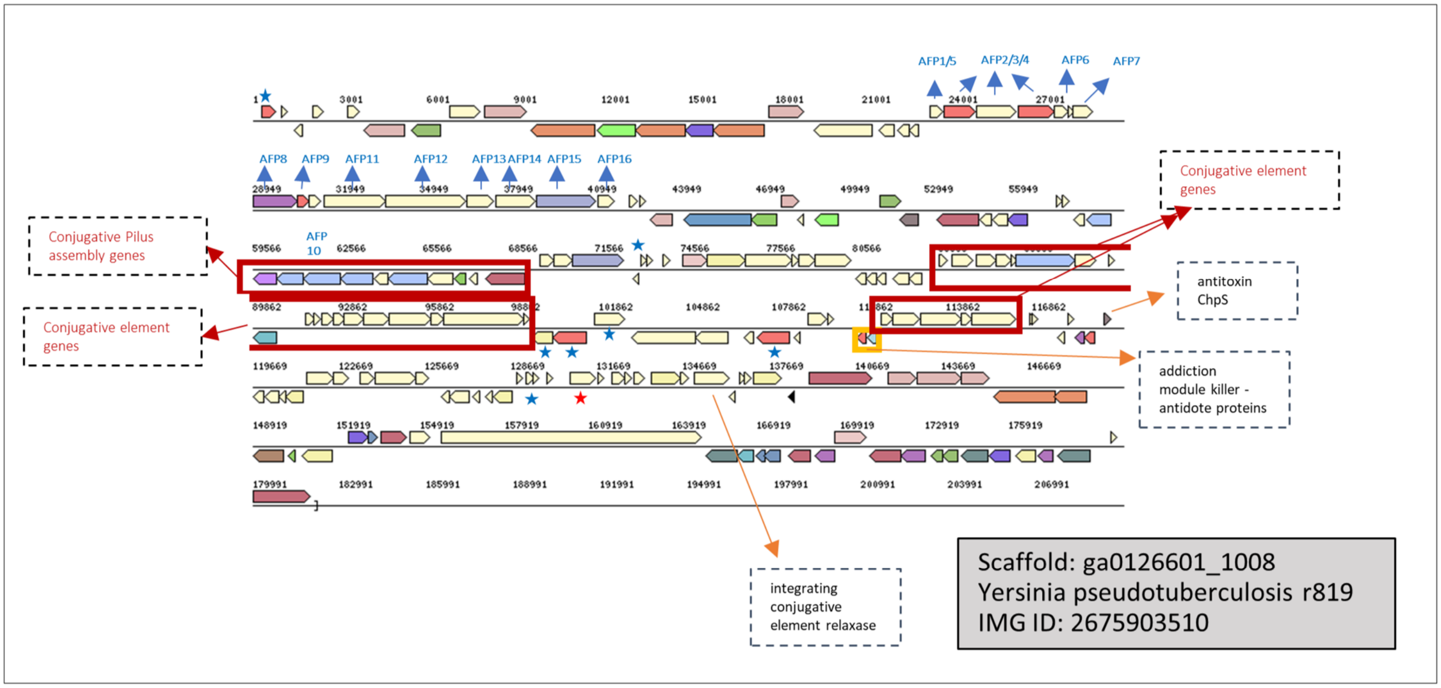
AFP1/5
AFP2/3/4
AFP6
AFP7
AFP8
AFP9
AFP11
AFP12
AFP13
AFP14
AFP15
AFP16
AFP 10
Conjugative Pilus assembly genes
Conjugative element genes
Conjugative element genes
antitoxin ChpS
addiction module killer - antidote proteins
integrating conjugative element relaxase
Scaffold: ga0126601_1008
Yersinia pseudotuberculosis r819
IMG ID: 2675903510

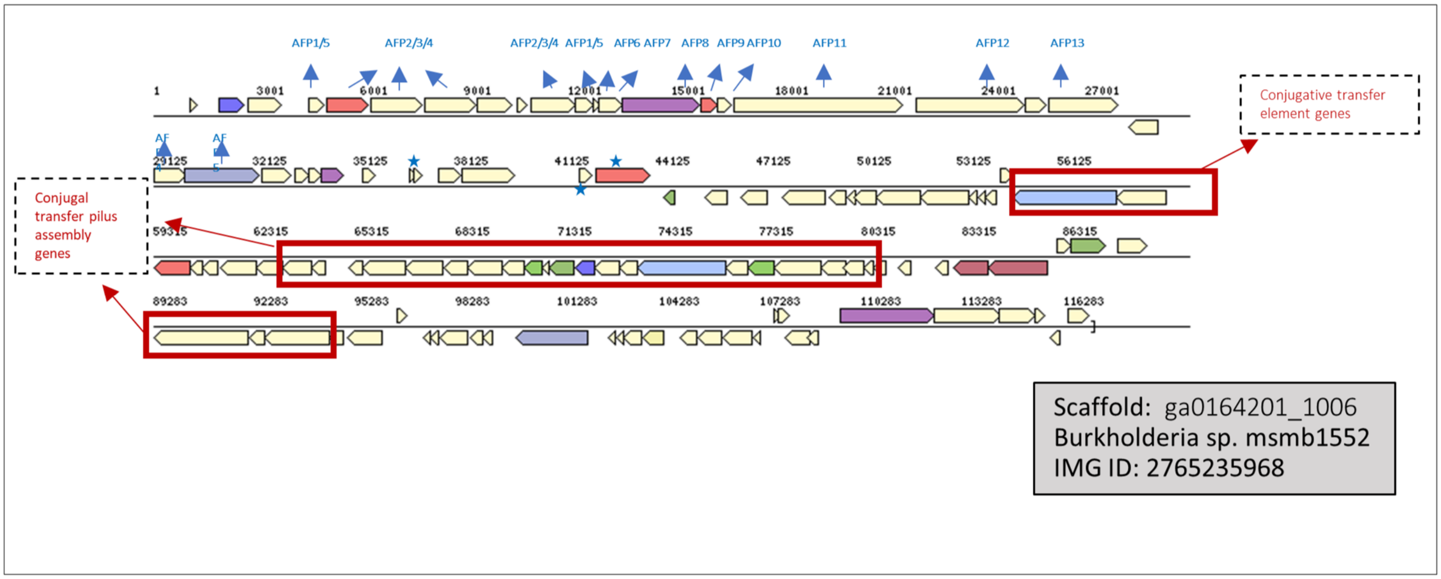
AFP1/5
AFP2/3/4
AFP2/3/4
AFP1/5
AFP6
AFP7
AFP8
AFP9
AFP10
AFP11
AFP12
AFP13
Conjugative transfer element genes
Conjugal transfer pilus assembly genes
Scaffold: ga0164201_1006
Burkholderia sp. msmb1552
IMG ID: 2765235968

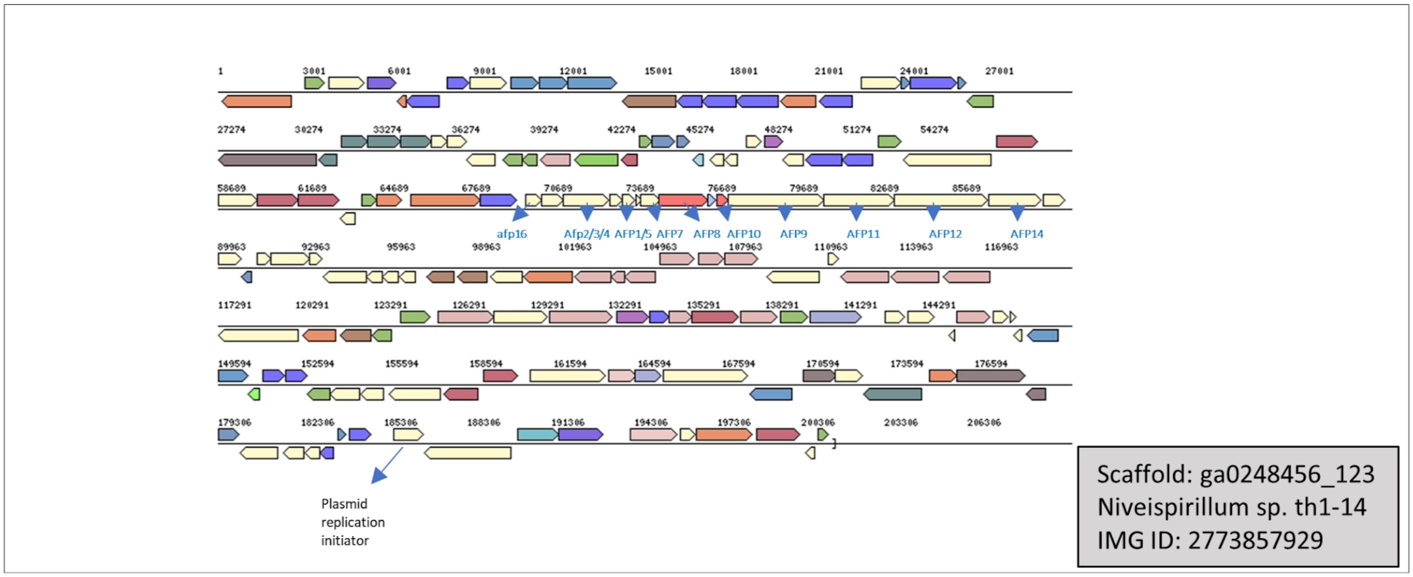

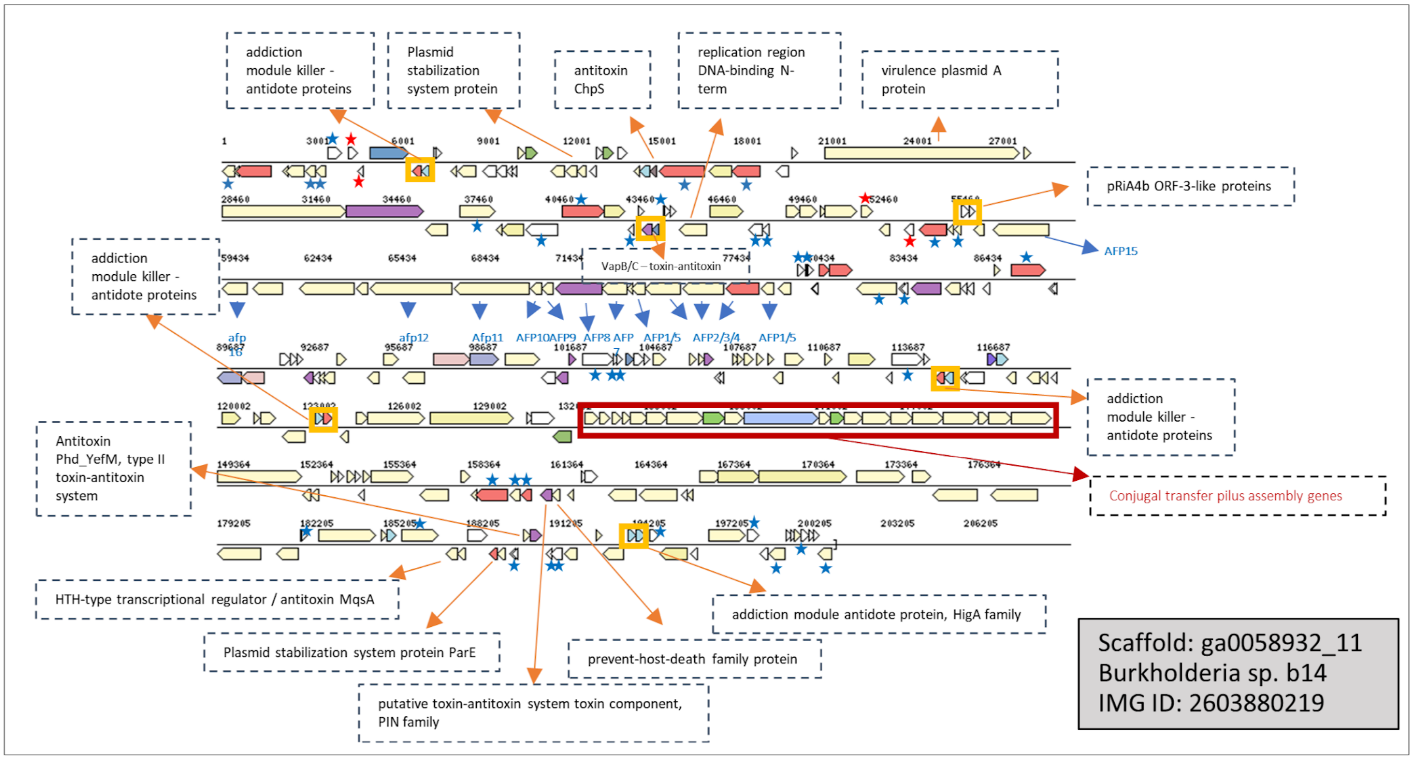

Scaffolds predicted to be eCIS containing plasmids

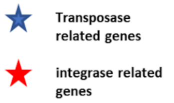

Supplementary Figure 6. Four examples of predicted plasmid-borne eCIS operons. IMG genome ID, Species name, and DNA scaffolds are listed in grey legend in bottom right of each image. Genes that play a role in plasmidic activities are marked with arrows pointing to their annotations in dashed boxes, e.g addition modules,

plasmid stabilization proteins, conjugative elements, etc. Blue arrows indicate eCIS operon. Red stars mark integrase-related genes, blue stars indicate transposase related genes.

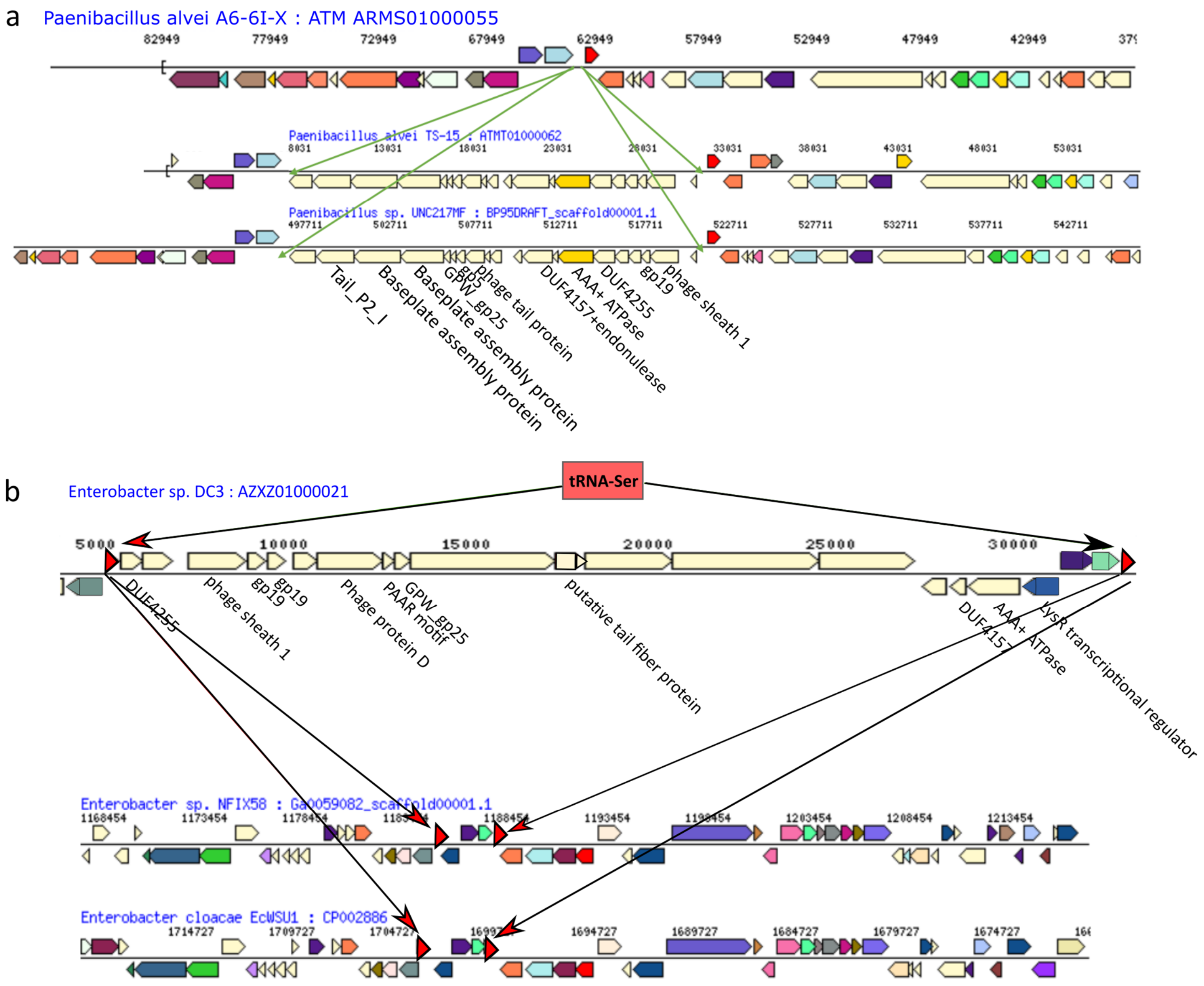

Supplementary Figure 7. Evidence for local eCIS locus integration events into bacterial genomes. a. A local eCIS integration into specific strains of *Paenibacillus* strain. b. A local eCIS insertion into an *Enterobacter* strain. The integration was likely mediated via homologous recombination using the flanking eCIS tRNA-Ser genes and an external eCIS copy that carries this gene.

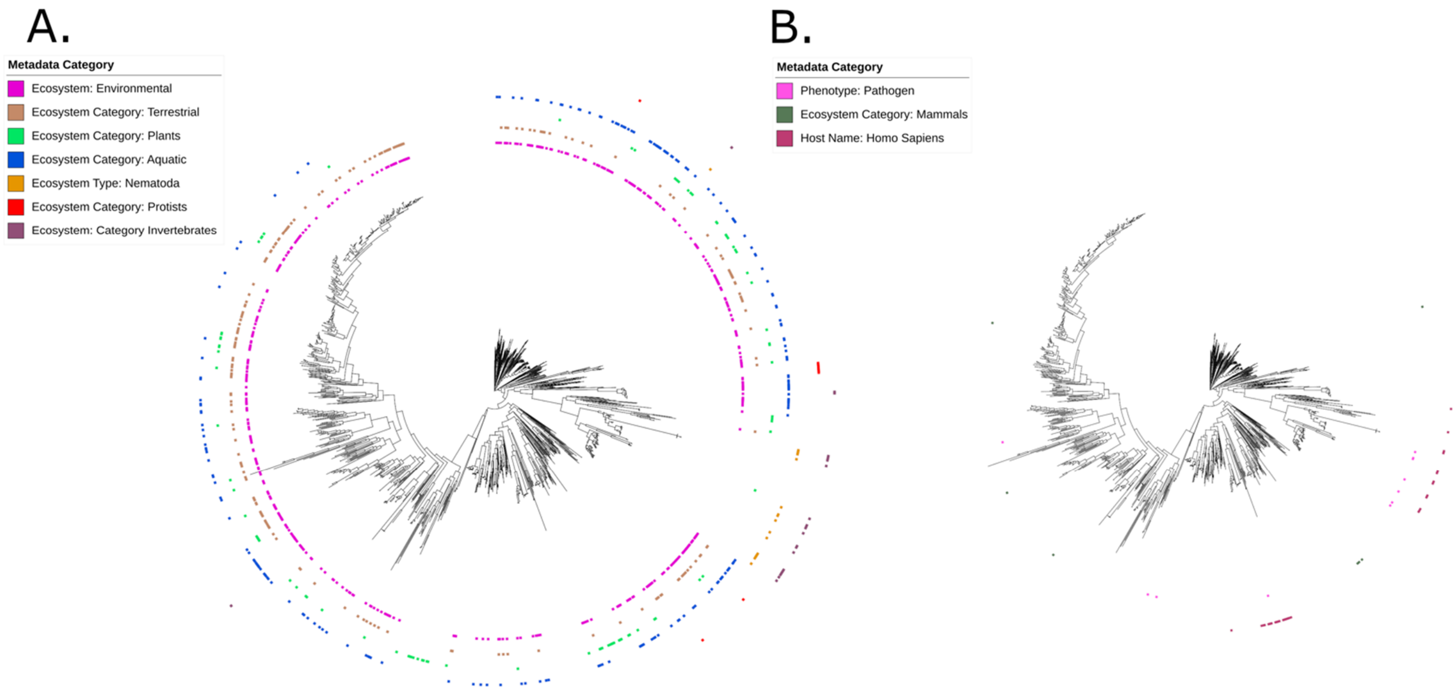

Supplementary Figure 8. eCIS correlation with different metadata of the eCIS-encoding microbe. Each genome containing eCIS has a filled square corresponding to the presence of metadata indicated in the key. Panel A displays a selection of categories that were enriched statistically in eCIS-encoding genomes. Panel B displays a selection of categories that are statistically depleted in eCIS-encoding genomes.

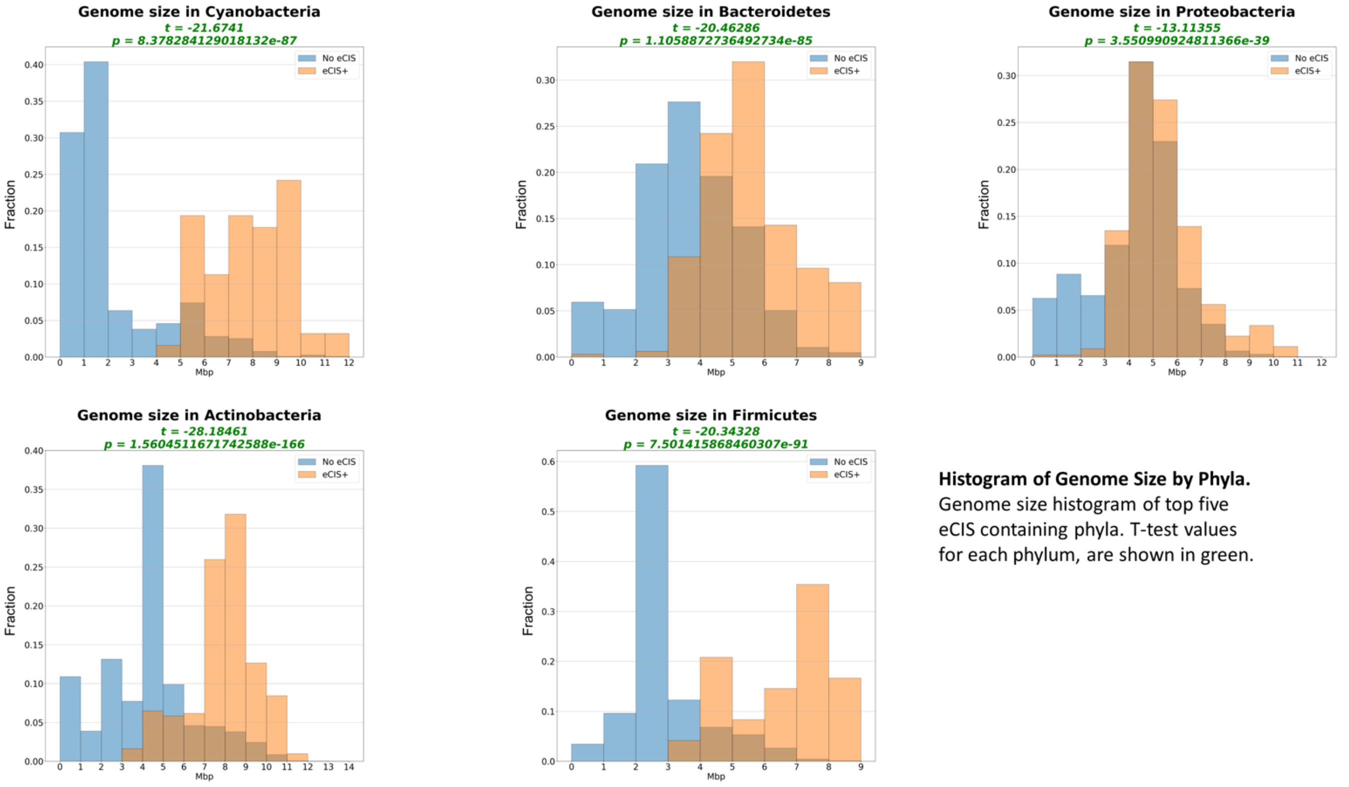

Supplementary Figure 9. Genome Sizes of eCIS-encoding versus non-encoding bacteria per Phylum. A normalized histogram shows genome size (X axis, binned in 1Mbp bins) and fraction of genomes in each bin (Y axis). Orange represents eCIS-encoding genomes, and blue represents genomes without eCIS. A two-sided t-test was carried out between each blue and orange distribution, and the statistics are listed above each graph in green.

<u>Query</u> <u>AFP 13 homolog -- IMG gene ID, genome name</u>
<u>Subject</u> <u>Organism name of BLAST hit in NCBI; Accession</u>

A.

Query Afp13 homolog = 637464290
(*Photorhabdus laumondii* TTO1)

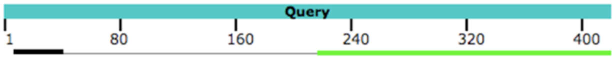

Subject = Organic Lake phycodnavirus; ADX06690.1

putative phage tail collar domain family protein [Organic Lake phycodnavirus]
Sequence ID: ADX06690.1 Length: 303 Number of Matches: 3

Range 1: 107 to 303 GenPept Graphics ▼ Next Match

Score	Expect	Method	Identities	Positives	Gaps
79.7 bits(195)	4e-21	Compositional matrix adjust.	72/227(32%)	103/227(45%)	55/227(24%)

```
Query  217  GLSLSKNGVKVKAGN---GIKVDDKG-------VSID--------PNKVLPRGMIVMFSG
            GL + K+     AGN    +K+D  G       V+ID         N  +PRG I+M+SG
Sbjct  107  GLGIDKDVALFDAGNEGQALKIDSVGDSHFYKDVNIDGNLTVSGVSNSFIPRGGIIMWSG

Query  259  SSVPEGWALCDGKDNRPNLIDRFIM-GGTTQNIGGKSSDSFSGAKDNKKFTFISESQTVR
              +PE W+LCDG +  P+L +RFI+  G++ NIG       SG          S ++T+
Sbjct  167  VIIPESWSLCDGTNGTPDLRNRFIVSSGSSYNIGT------SGG---------STTRTLS

Query  318  ISG-STDGHGLTADENGPHQHEQGETLNRQGKCHNGYTEDNTDRDWVDGGRRGPEPP---
            +S   +  H  T + +G H H Q            GY +   D+++   G  G  PP
Sbjct  212  VSNLPSHNHTGTTNNDGAHHHSQRL----------GYID---DKNFT--GHNGQRPPGDS

Query  374  -NYRAYTFPSGKGN-PHSHPINLTSGGHNHSNNVTVPYYILAFIIKL  418
             N R  T+     N  H H     S G   S ++  PYY LAFI+KL
Sbjct  257  GNVRTSTYTIDNTNSSHQHAFTTNSTGSGTSFDIRPPYYALAFIMKL  303
```

B.

Query Afp13 homolog = 2558033937
(*Photorhabdus temperata* J3)

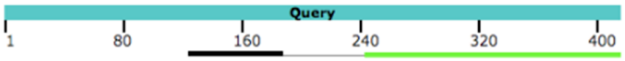

Subject = Yasminevirus sp. GU-2018; VBB19014.1

hypothetical protein YASMINEVIRUS_1546 [Yasminevirus sp. GU-2018]
Sequence ID: VBB19014.1 Length: 226 Number of Matches: 2

Range 1: 98 to 226 GenPept Graphics ▼ Next Match

Score	Expect	Method	Identities	Positives	Gaps
74.3 bits(181)	6e-20	Compositional matrix adjust.	54/176(31%)	78/176(44%)	51/176(28%)

```
Query  243  VLPRGMIVMFSGSSVPIGWALCNGENDTPNLIDRFIMGGTTQDIGGQSSNSFSGSKKDKK
            VLPRG+IV +SGS++P GWALC+G N TPNLIDRFI+G   ++         + +
Sbjct  98   VLPRGIIVAWSGSTIPNGWALCDGSNGTPNLIDRFILGSRIEN---------AKATGGSS

Query  303  FTFTSENQTVHISGKTDGHGLTADENGKHTHEQGETLNEKGMCHNKYTTDTSSRDWVNGG
             T  +E Q                    H H+     +  G+C  K    +    + +GG
Sbjct  149  TTTLTEAQM-----------------PPHKHDMMHKDHPAGICDVKNCGKSDGAFYASGG

Query  363  SSGSNPPKYRPYTFPSGE----GKPHSHSINLTSGEHSHKNNVTVPYYILAFIMKL  414
            +S      YR +  P+ E    G+P S               +  P+Y LAFIM+L
Sbjct  192  AS------YRLFNRPTTESAGGGQPFS---------------IMPPFYKLAFIMRL  226
```

C.

Query Afp13 homolog = 2523973452
(*Aquimarina latercula* DSM 2041)

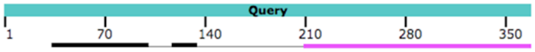

Subject = Burkholderia virus Bcep22l; NP_944294.2

NP_944294.2 phage tail fiber protein [Burkholderia virus Bcep22]
Sequence ID: Query_58581 Length: 292 Number of Matches: 3

Range 1: 118 to 290 Graphics ▼ Next Match

Score	Expect	Method	Identities	Positives	Gaps
96.7 bits(239)	2e-27	Compositional matrix adjust.	68/180(38%)	86/180(47%)	28/180(15%)

```
Query  210  GLISMWSGQ--DIPK----GWVLCDGTNNTPDLSGKFIVGMEKNNPEYQIGKKGGAKEVT
            G + +W+G   DIP     GW L DG N T DL  KFIV    +   Y  G  GGA  V
Sbjct  118  GDLKIWTGAITDIPAVHGPGWYLADGQNGTIDLRDKFIVAAGGS---YAPGNTGGAATVA

Query  264  LTEAQMPSHTHL--DKGHNHNITDPGHNHNHGSFNGLVRFTGRDTTDTMDNNPGAGKEFA
            LT AQMP H H   D GH H ++DP H H   S        G +T     ++ G G  F
Sbjct  175  LTVAQMPQHNHGVNDPGHGHGVSDPTHAH---SVYDPGHSHGHNTAALTPSSTGGGA-FQ

Query  322  LNNF--GTIQSNKTGVSIKTAKANL-----------QSTGGNKAHENRPPYFVLAYIMKL
            +N +  GTI ++ TG+ I  A   +           Q+ G   AHENRPPY+ LA I  +
Sbjct  231  INGYAGGTINASATGIGIYGAATGISIQGAGTGISTQNAGSGTAHENRPPYYALAVIQYV
```

D.

Query Afp13 homolog = 2599844965
(*Thiothrix caldifontis* DSM 21228)

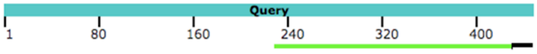

Subject = Staphylococcus phage vB_SauS_IMEP5; ANM47011.1

tail fiber protein [Staphylococcus phage vB_SauS_IMEP5]
Sequence ID: ANM47011.1 Length: 603 Number of Matches: 3

Range 1: 396 to 601 GenPept Graphics ▼ Next Match

Score	Expect	Method	Identities	Positives	Gaps
72.4 bits(176)	8e-18	Compositional matrix adjust.	64/215(30%)	95/215(44%)	25/215(11%)

```
Query  228  WSSLSFNAHHNENNNAWVFPDNTTTAVTLEMDHSTG-LNQARFDVWTTTTQSKTAWALRL
            +S  S+   H     A +  D T T  TL  D S G  N   F+       S    A+R
Sbjct  396  FSRCSYGTVH-----ACLMDDGTITKPTLVWDRSGGGFNSCHFNNQRQCANSMNGSAIRC

Query  287  RILGDSGNV--GIGVEQPSEKLEVNGNIK-----------INGGIKTNFITQEDWIAPTL
                  G++   IGV   + ++  NG I+           + GG+   + T+ D    T
Sbjct  451  DSTNTHGSIKSDIGVVVQAAEIFYNGTIEWIEGATRPESYVQGGMINKYATEIDL---TT

Query  334  LNGWVYYGHVWNTAGYFKDSFGIVHLKGLIKNGS--TGQPIFTLPAGYRPAQQELHAACT
             NGW  Y   +      +   G VHL GLIK+G+  +G   FTLP GY P  + ++
Sbjct  508  KNGWNSYSADYRPRA-LRMPDGTVHLYGLIKDGTPGSGSVAFTLPKGYTPKTKGIYGGFV

Query  392  NSNTIGRIDIRANGEVIMESGSNAWISLDGITFRA  426
            +  +IG+I I  +G   +ES +  +ISL GI+F A
Sbjct  567  SDGSIGKILIDVSGNFAVESMTGQYISLSGISFYA  601
```

E.

Query Afp13 homolog = 2640816185
(*Photorhabdus thracensis*:DSM 15199)

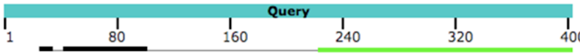

Subject = Phage 5P_1; AZF90167.1

hypothetical protein BPH43C_44 [Phage 5P_1]
Sequence ID: AZF90167.1 Length: 237 Number of Matches: 4

Range 1: 86 to 237 GenPept Graphics ▼ Next Match

Score	Expect	Method	Identities	Positives	Gaps
70.5 bits(171)	1e-18	Compositional matrix adjust.	59/184(32%)	84/184(45%)	35/184(19%)

```
Query  224  LPKGMIVMFSGSSAPTGWAFCDGKTYNGVTVPDLRSRFVMCGETISDTGKSSNKASGGSS
            +P G+I+++SG + P+GWA CDG   NG   PDLR+RFV+        G+       G+
Sbjct  86   VPSGLIILWSGQNVPSGWALCDGS--NG--TPDLRNRFVIGAGGDHSPGQIGRPIPEGTQ

Query  284  AKNFFRDTKPTTVSVSVNVQNTTLTEAQIPSHKHIGGMPYCWGTGMKYIGFSD---TQTQ
                    KP T +V++   + TL E  + +H +    P    TG     +     T T
Sbjct  142  ----LYQIKP-TATVTIGGHSLTLAEV-MHTHSYYDYTP----TGNTLGAYGSRRVTSTT

Query  341  YQIDNTYNSSIWRKHSDGQSLYACTSNTGGGQGHNHHATASSPSHNHSVDVIPPYYLLAF
            Y +  T               YAC + +GG   H H AT +     +  DV PPYY LA+
Sbjct  192  YIVRAT--------------GYACGNASGGADAHTHSATFT----GNDFDVRPPYYKLAY

Query  401  IMKL  404
            IMKL
Sbjct  234  IMKL  237
```

F.

Query Afp13 homolog = 2609602034
(*Chitinophaga* sp. CF418)

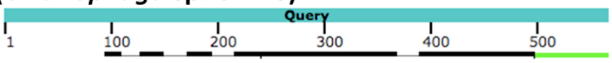

Subject = uncultured Mediterranean phage uvMED; BAR33157.1

virion structural protein [uncultured Mediterranean phage uvMED]
Sequence ID: BAR33157.1 Length: 1620 Number of Matches: 13

Range 1: 1514 to 1620 GenPept Graphics ▼ Next Match

Score	Expect	Method	Identities	Positives	Gaps
75.1 bits(183)	9e-18	Compositional matrix adjust.	45/113(40%)	66/113(58%)	7/113(6%)

```
Query  452   KGKARAFGLYLD-TDVRKVSNPVKLGNVIDKVKKLNPVTFTFNQKANCPPAEAQIGFLPH
             + +  A G ++D +DV    + V +   +D VKKL P T+        P  E QIGF+
Sbjct  1514  QAQLSAAGAWVDASDVAYKKDIVDINYGLDTVKKLKPRTYKMK-----PDDEQQIGFVAQ

Query  511   QVEEFFPELVNTDGDGTQTLAYANMVAVLTKAIQEQQETIAALQKRLEALEGK  563
             ++E   PE+V+ + DG++ +AY  + AVLTKAIQEQQE I  LQ ++  L GK
Sbjct  1569  ELELDIPEIVSGE-DGSKGVAYGQLTAVLTKAIQEQQELIEDLQTQINNLRGK  1620
```

Supplementary Figure 10. Examples of high identity BLAST protein sequence alignments between Afp13 sequence queries and tail fiber proteins from bacteriophages and viruses as the targets. A and B show hits to eukaryotic viruses, C-F show hits to bacteriophages.

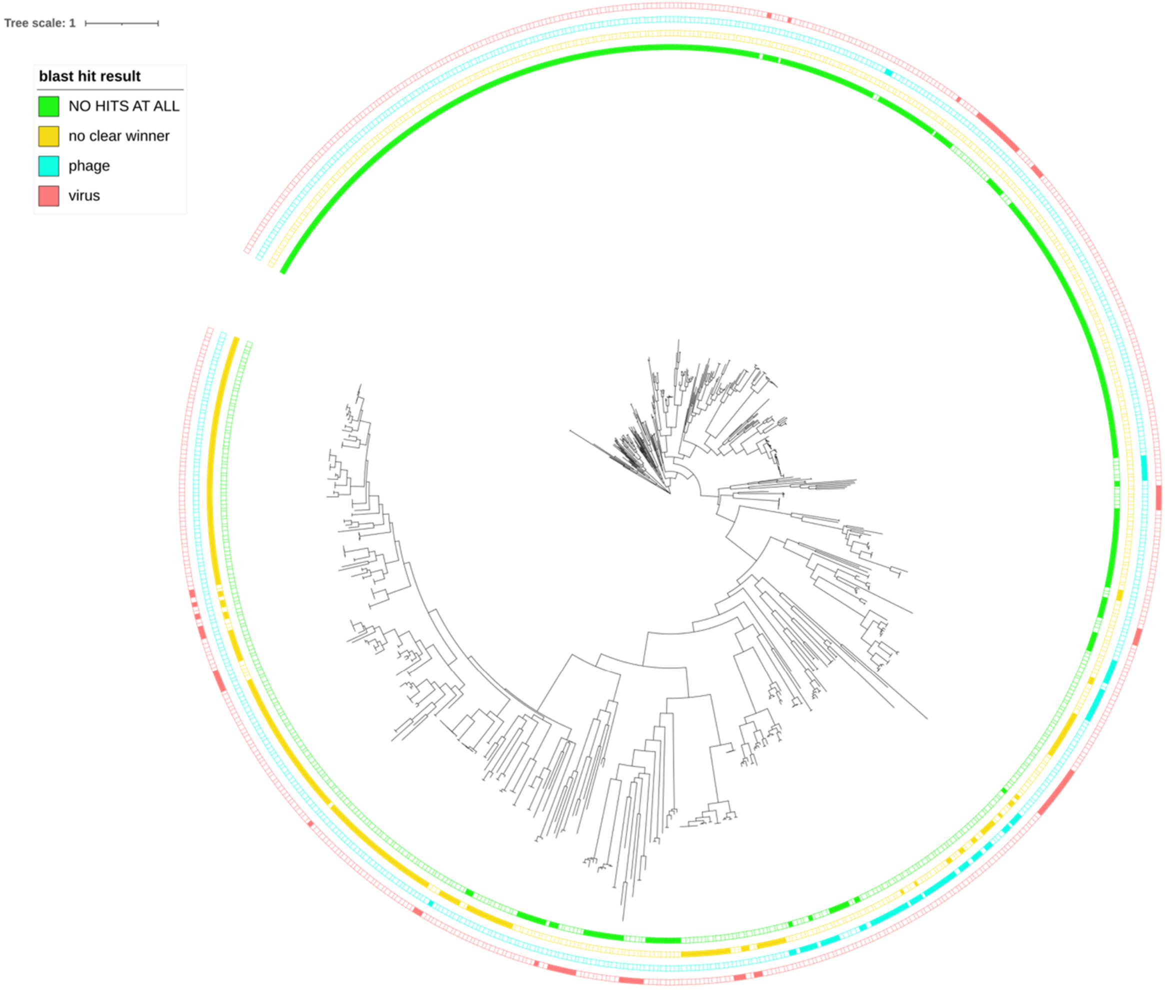

Supplementary Figure 11. Phylogenetic relationship of tail fiber genes. Maximum likelihood tree of tail fiber genes. The filled squares in the rings correspond to the structural similarity (best blast hit) of each Afp13, as described in the key.

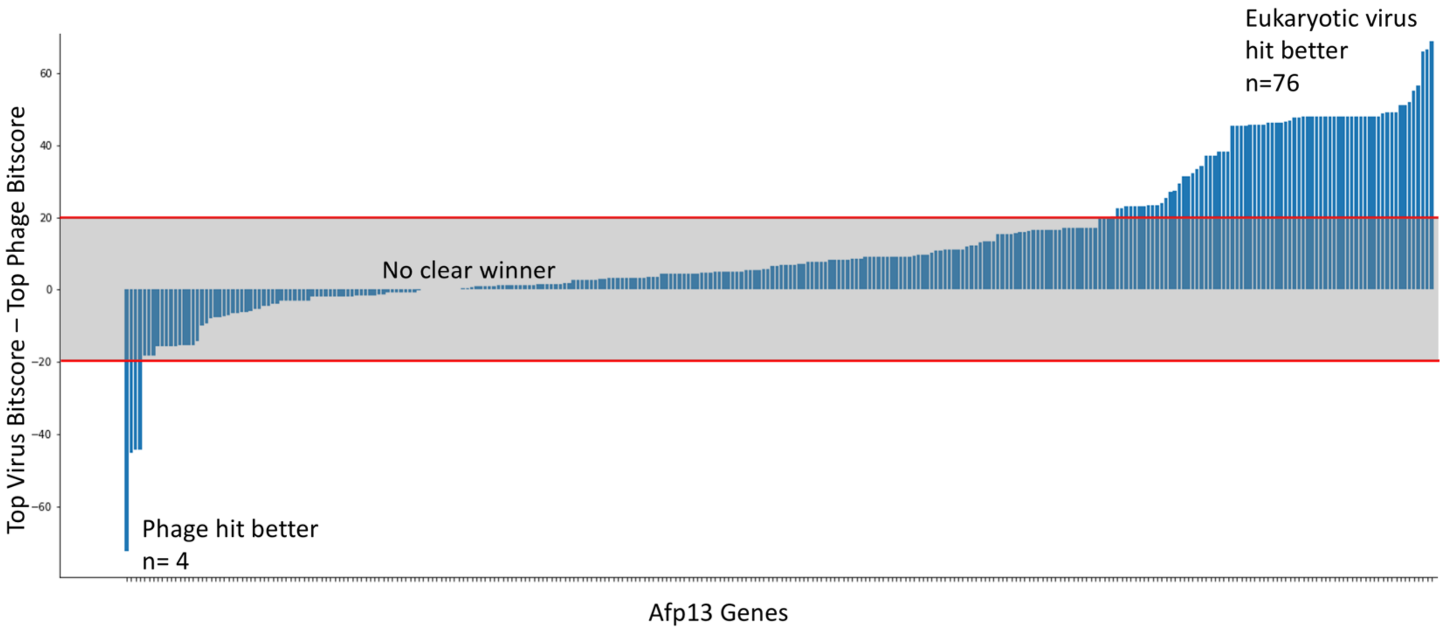

Supplementary Figure 12. Afp13 blast bitscores. X-axis shows Afp13 genes. Afp13 genes top bitscore versus a phage database was subtracted from its best hit against a Eukaryotic-virus targeting database, and displayed on the Y-axis. Red lines indicate cutoff values for where Afp13 were considered to have comparable similarity to both phage and Eukaryotic-targeting viruses (grey area, "No clear winner").

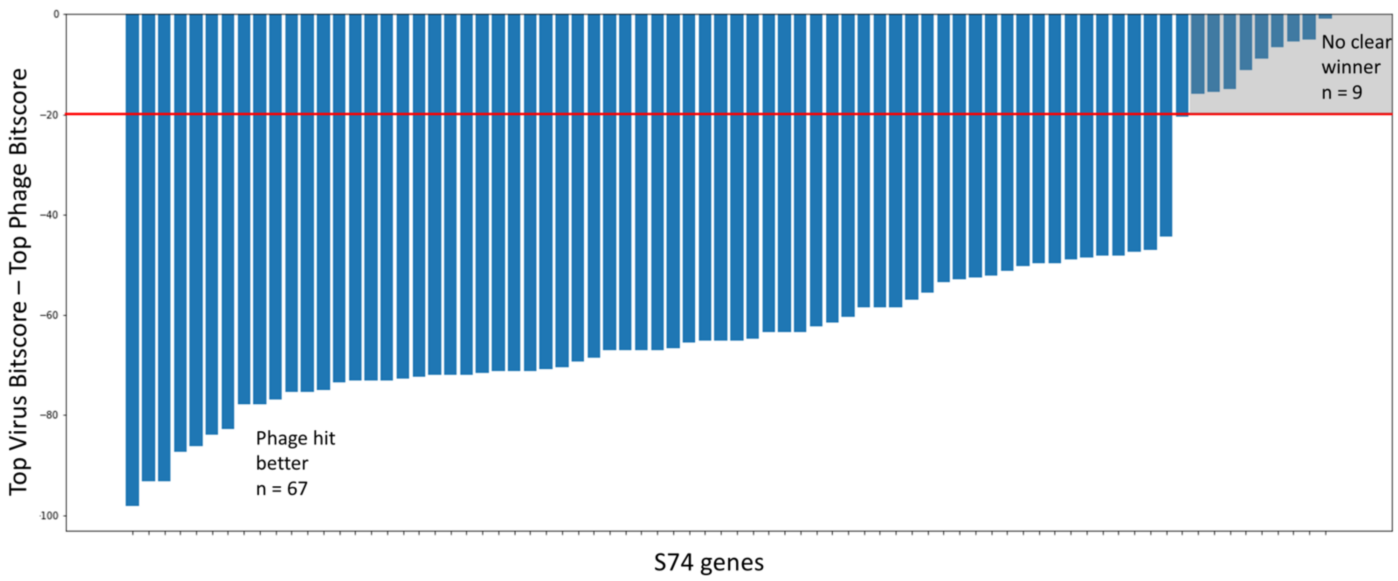

Supplementary Figure 13. Peptidase S74-containing genes. Peptidase S74-containing genes top bitscore versus a phage database was subtracted from its best hit against a Eukaryotic-virus targeting database, and displayed on the Y-axis. Red line indicates indicate cutoff values for where Peptidase S74-containing genes were considered to have comparable similarity to both phage and Eukaryotic-targeting viruses (grey area, "No clear winner").

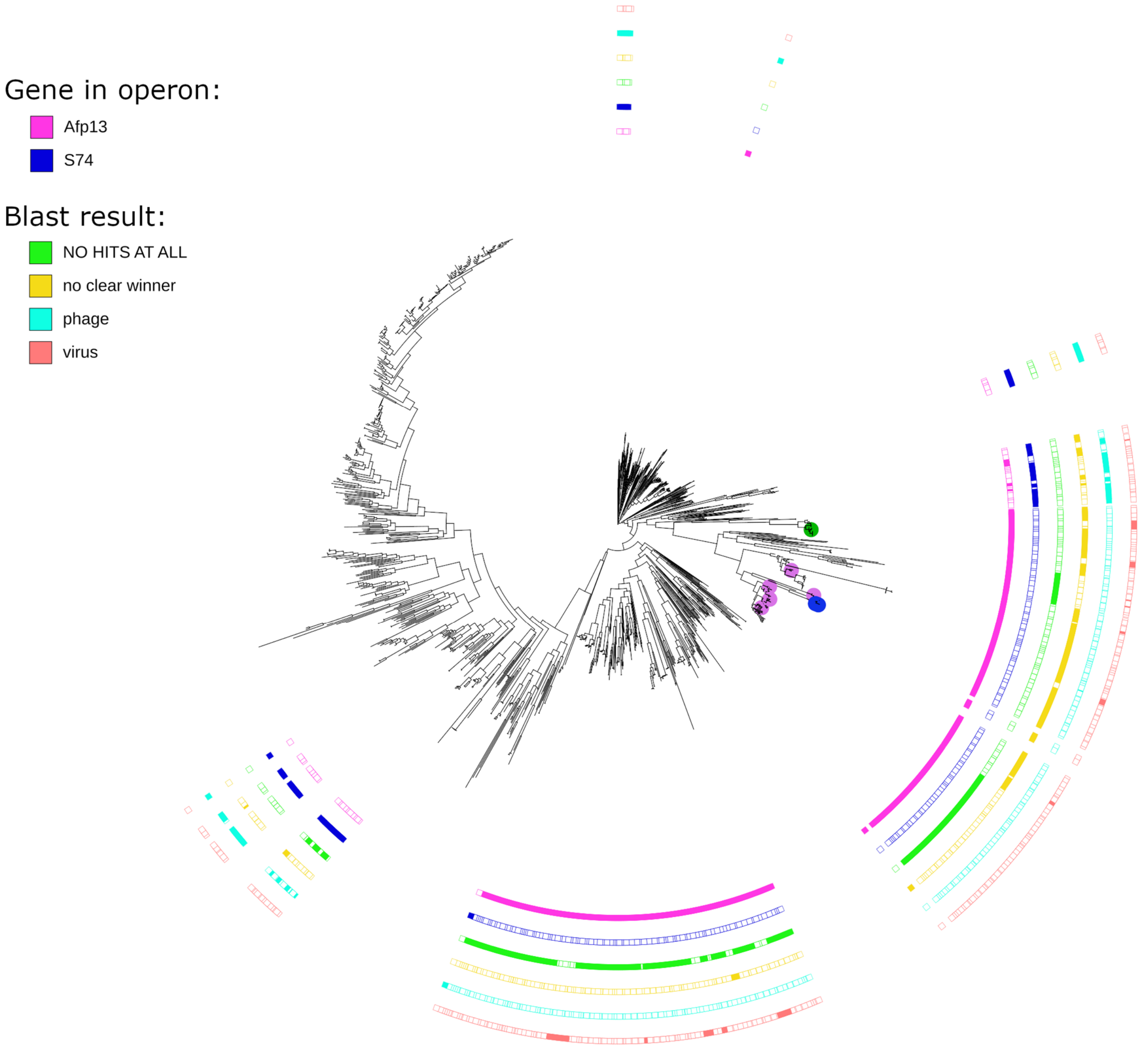

Supplementary Figure 14. Distribution of Afp13 and S74 presence/absence patterns across the eCIS tree and the hit distribution of Afp13 genes based on BLASTP analysis against phage and anti-eukaryotic viral genes. A filled in color corresponds to True, while an empty frame corresponds to False. Colors are described in the key.

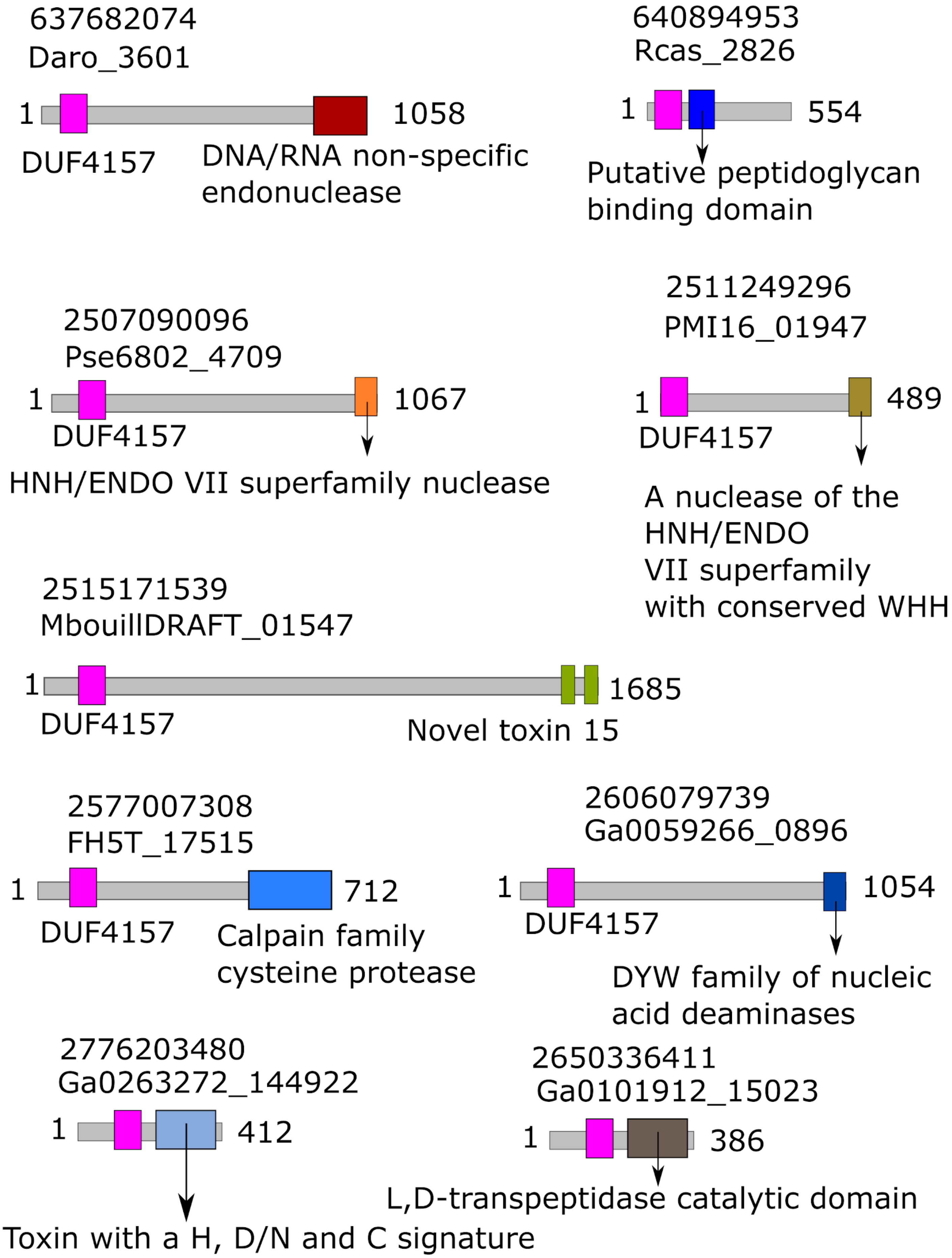
637682074
Daro_3601
1
1058
DUF4157
DNA/RNA non-specific endonuclease
640894953
Rcas_2826
1
554
Putative peptidoglycan binding domain
2507090096
Pse6802_4709
1
1067
DUF4157
HNH/ENDO VII superfamily nuclease
2511249296
PMI16_01947
1
489
DUF4157
A nuclease of the HNH/ENDO VII superfamily with conserved WHH
2515171539
MbouillDRAFT_01547
1
1685
DUF4157
Novel toxin 15
2577007308
FH5T_17515
1
712
DUF4157
Calpain family cysteine protease
2606079739
Ga0059266_0896
1
1054
DUF4157
DYW family of nucleic acid deaminases
2776203480
Ga0263272_144922
1
412
Toxin with a H, D/N and C signature
2650336411
Ga0101912_15023
1
386
L,D-transpeptidase catalytic domain

Supplementary Figure 15. Examples for putative eCIS-associated toxins (EATs) carrying DUF4157 at the N terminus. The upper number indicates IMG gene ID. The lower number indicates the locus tag (from IMG database).

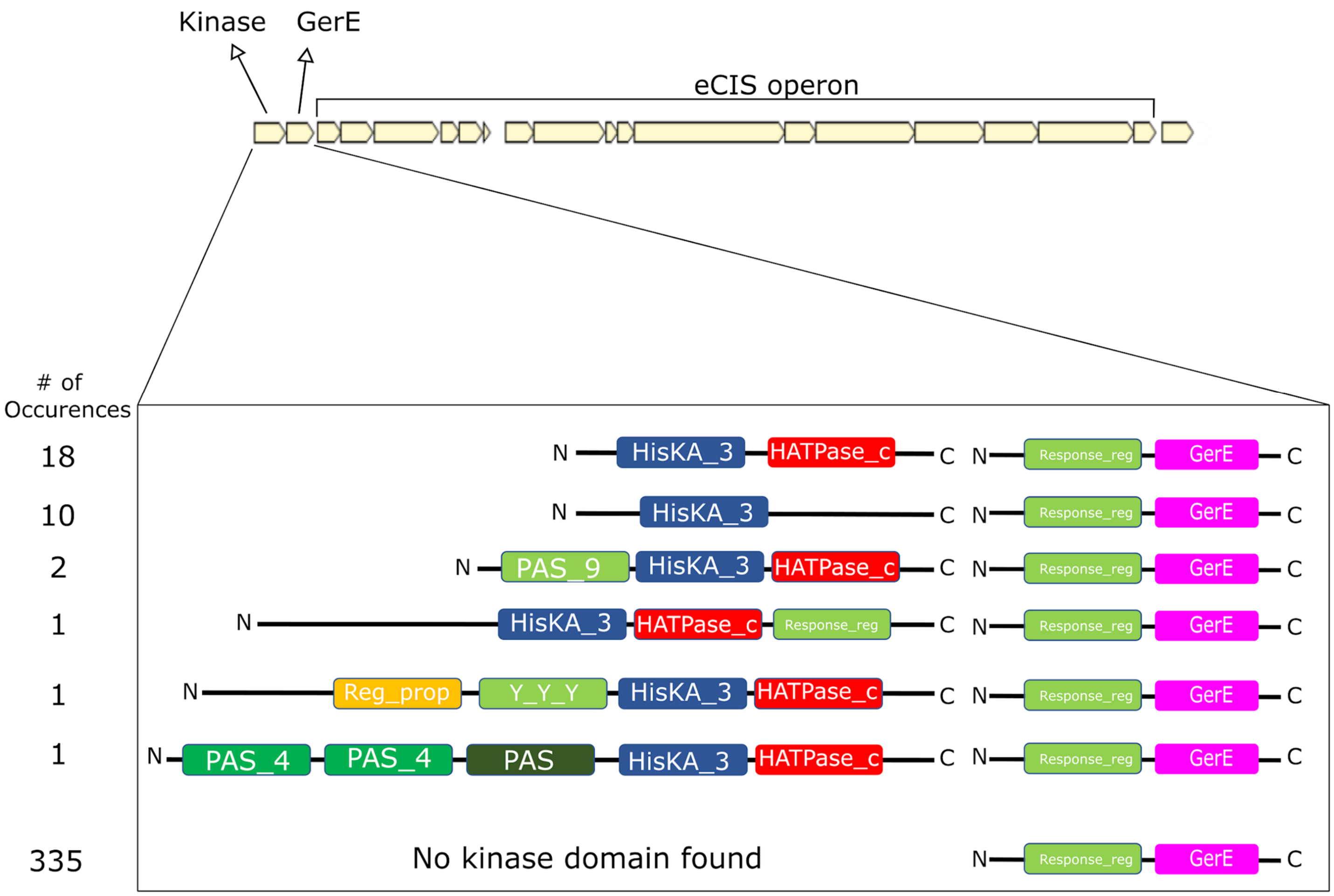

Supplementary Figure 16. Kinase domains are found upstream to the putative GerE eCIS transcription regulator. An illustration of the different pfam domain architectures and their occurrences within the kinase genes found upstream to the GerE gene that is found in the eCIS operons.

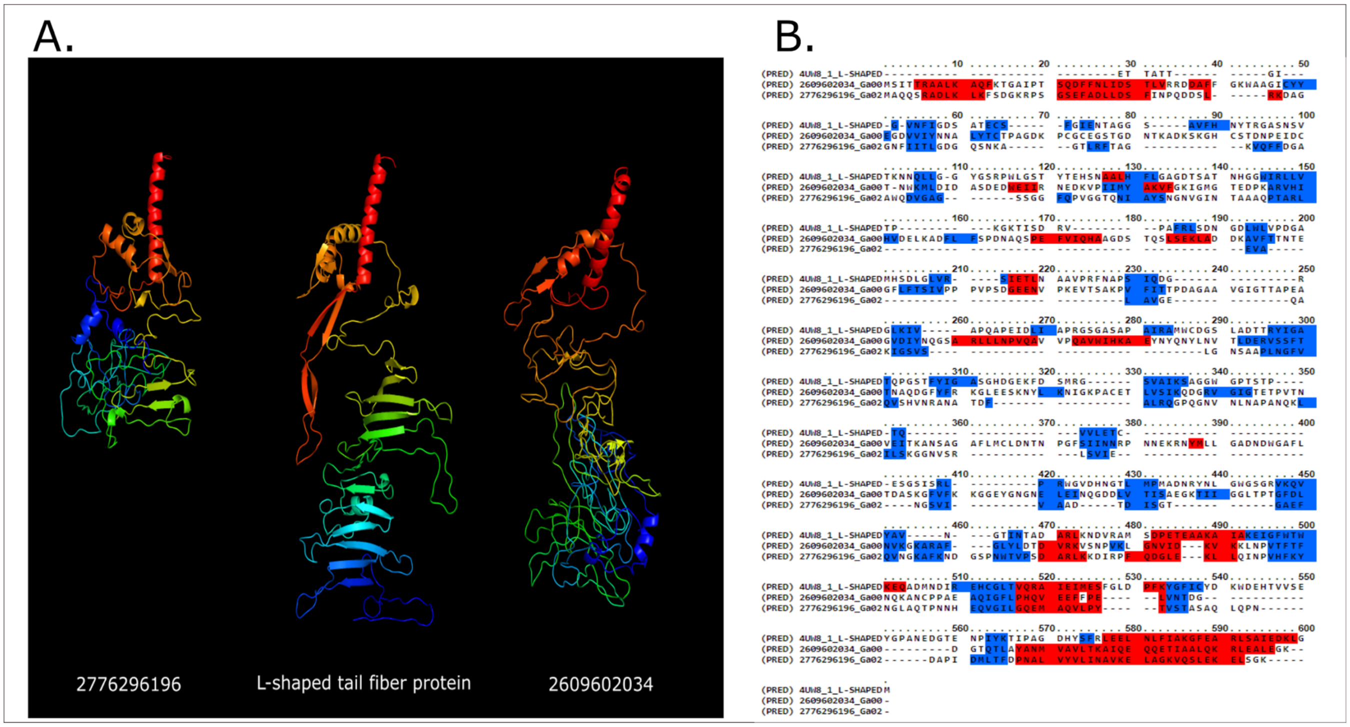

Supplementary Figure 17. Genes containing S_74 peptidase domain structurally resemble the carboxy-terminal domain of the T5 Phage tail fiber. A. 3D structure of cluster representatives (IMG: 2609602034, 2776296196) containing the S_74 Peptidase domain, compared to the carboxy terminal domain of the T5 tail fiber (PDB accession: 4UW8), predicted by Phyre2 with intensive modelling mode[75]. B. Alignment of the secondary structure with the T5 phage L-shaped tail fiber (PDB accession: 4UW8) using Praline [78].

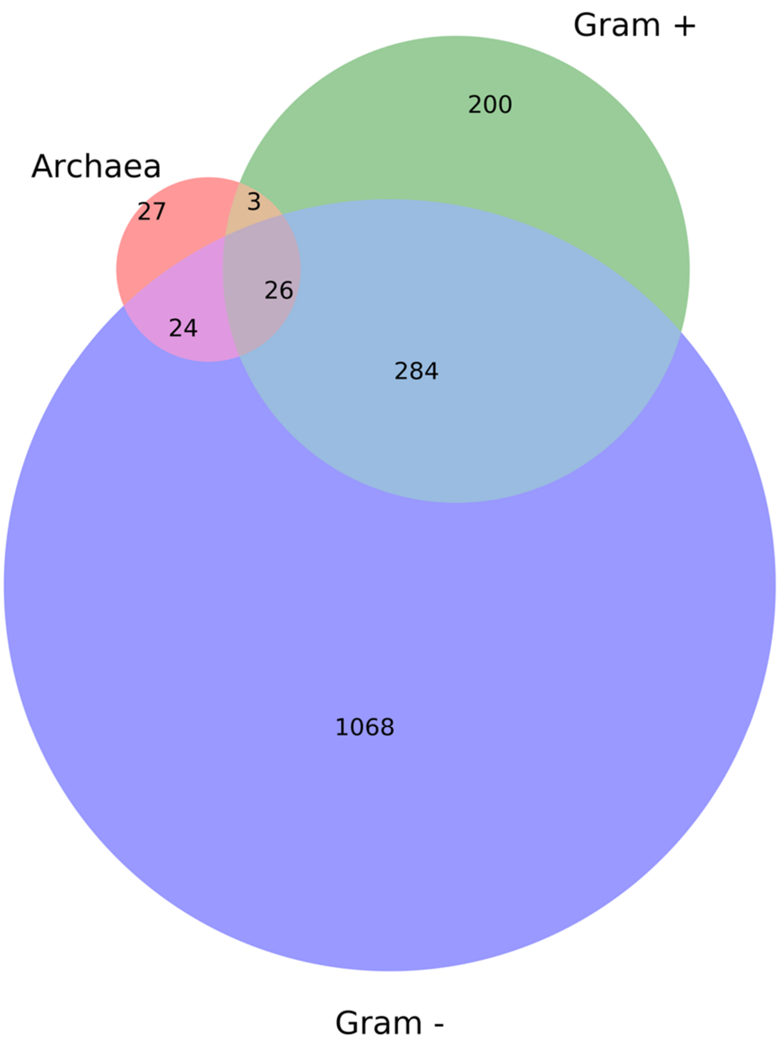

Supplementary Figure 18. Venn diagram of pfam domains in eCIS-containing genomes. Number of unique pfam domains found in eCIS-containing genomes from each of the following groups: Archaea, Gram positive, and Gram negative. Domains associated with core eCIS components shared by all groups are in the center of the Venn diagram.

Hemopexin (PF00045)

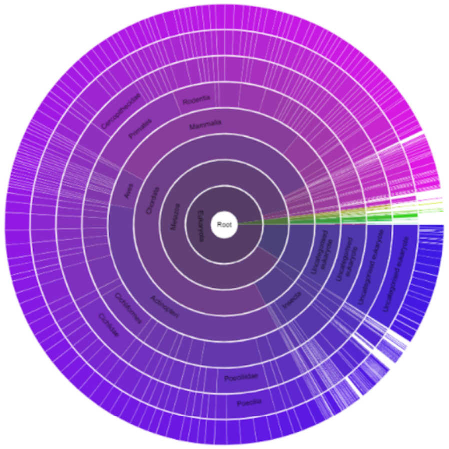

Annexin (PF00191)

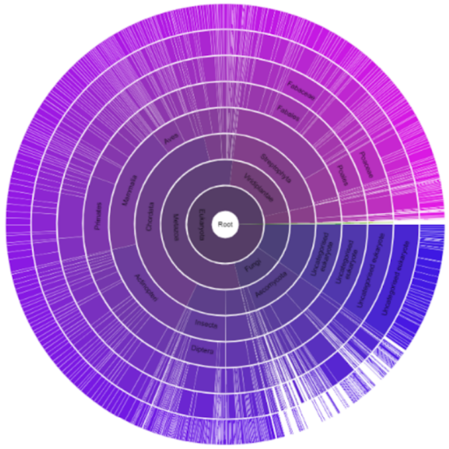

MORN (PF02493)

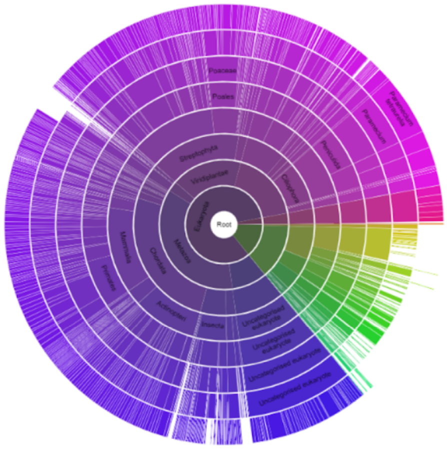

Prominin (PF05478)

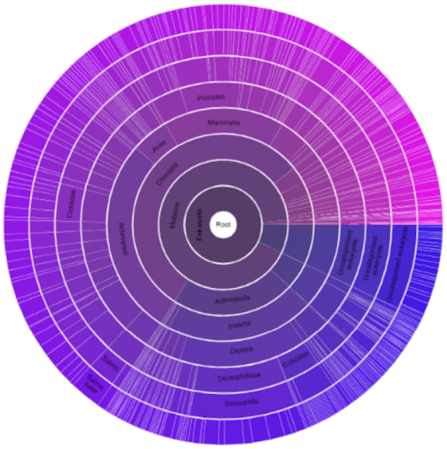

GRP (PF07172)

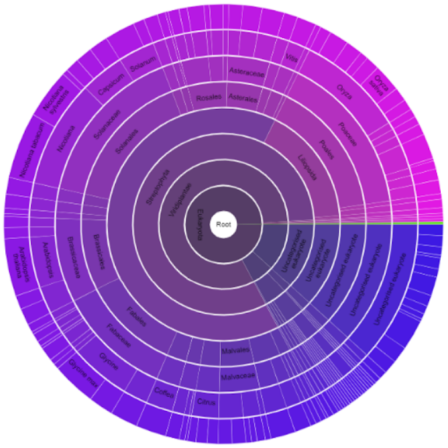

PAAR_motif (PF05488)

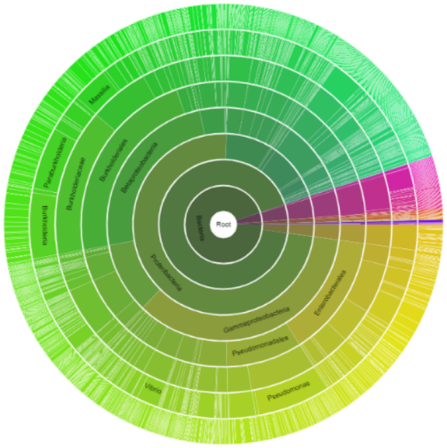

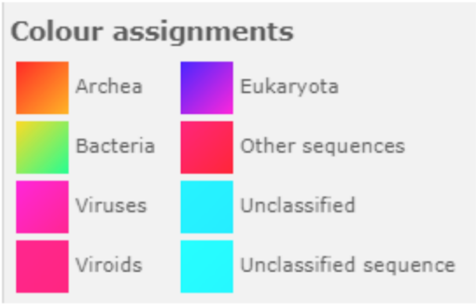

Supplementary Figure 19. Several eCIS toxins carry protein domains that are mostly found in eukaryotic annotated genomes. The sunburst plots, representing species distribution and taken from Pfam website (https://pfam.xfam.org/), represent the distribution of protein domains in available annotated genomes. The color coding for each plot appears below. As a reference we added the PAAR motif found in the eCIS spike which is mostly a bacterial domain.

All antibacterial EATs repeat

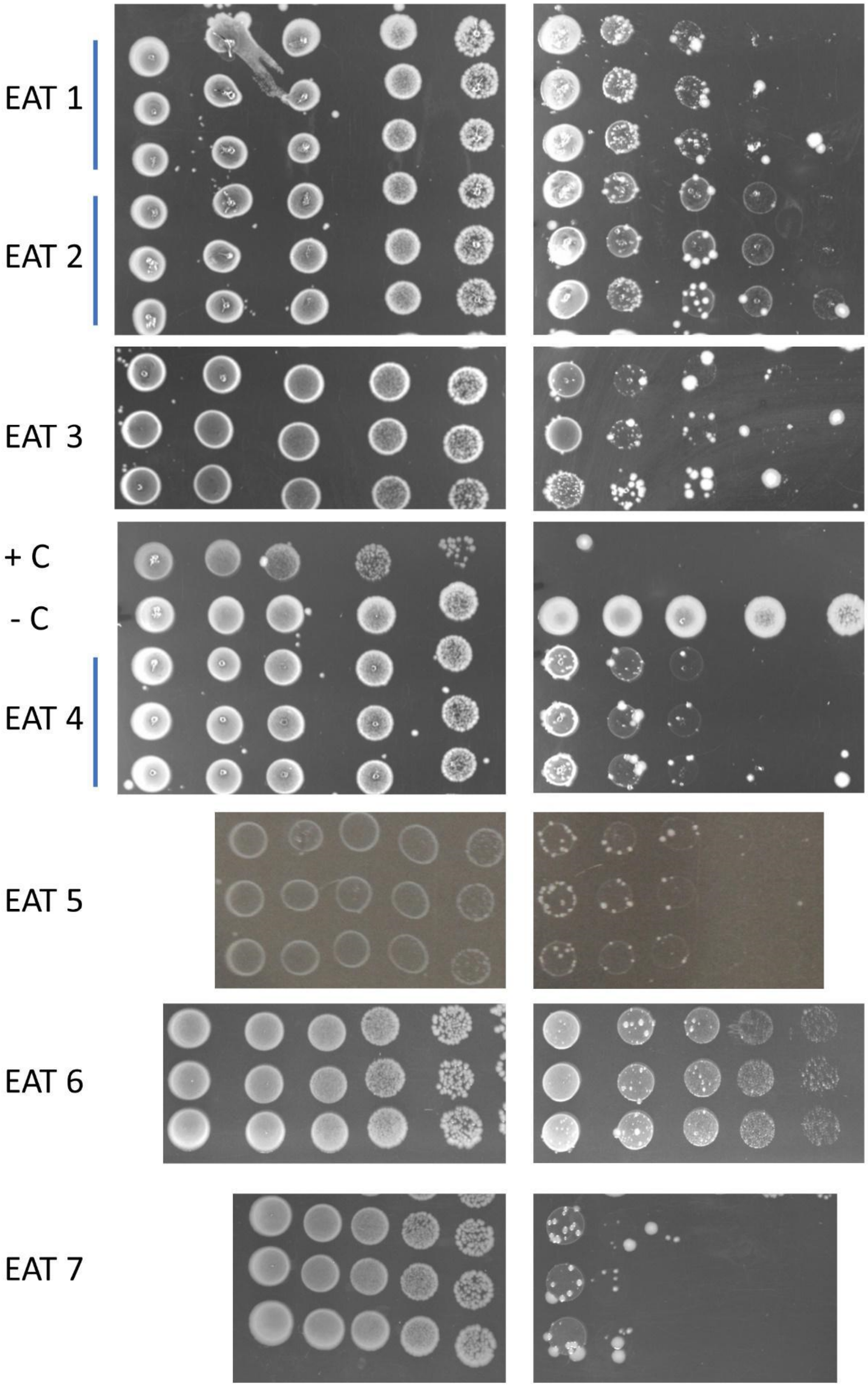

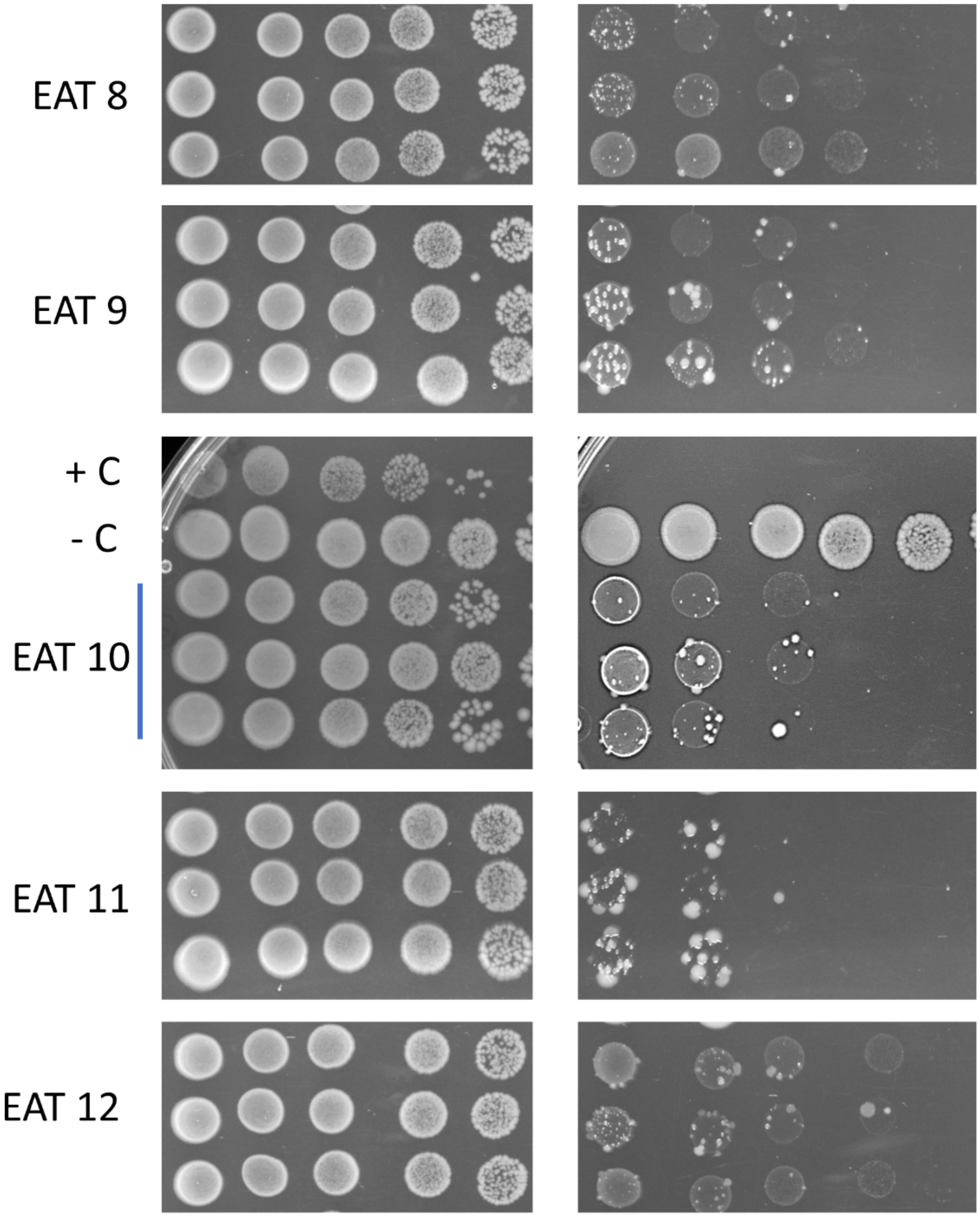

Supplementary Figure 20. Antibacterial EAT biological replicates. Drop assays that correspond to Figure 4 are shown here in full with three biological replicates each. "+ C", positive control; "- C", negative control. Left panels are without IPTG induction; right panels are with IPTG induction.

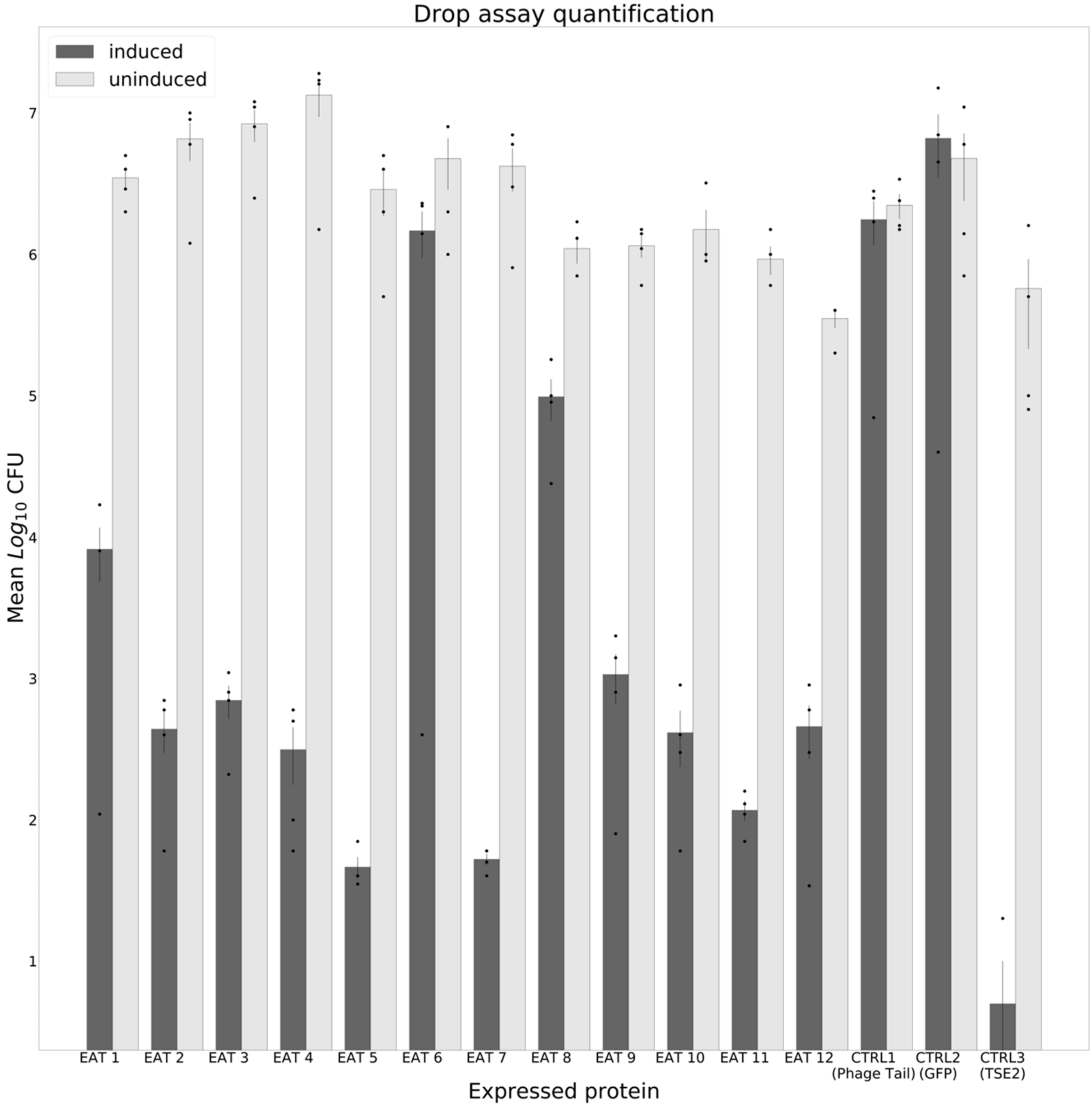

Supplementary Figure 21. Drop assay quantification. X-axis indicates the tested gene; Y-axis indicates the mean $\log_{10}$(CFU) of each *E. coli* strain not expressing (grey) and expressing (black) the given gene. Error bars display the standard error of the mean. Note that for expressed genes where there is only mild decrease in cfu, such as EAT6, the colonies are much smaller in the induced state (Supplementary Figure 20). n = 4 independent colonies per expressed protein, observed in 2 independent experiments.

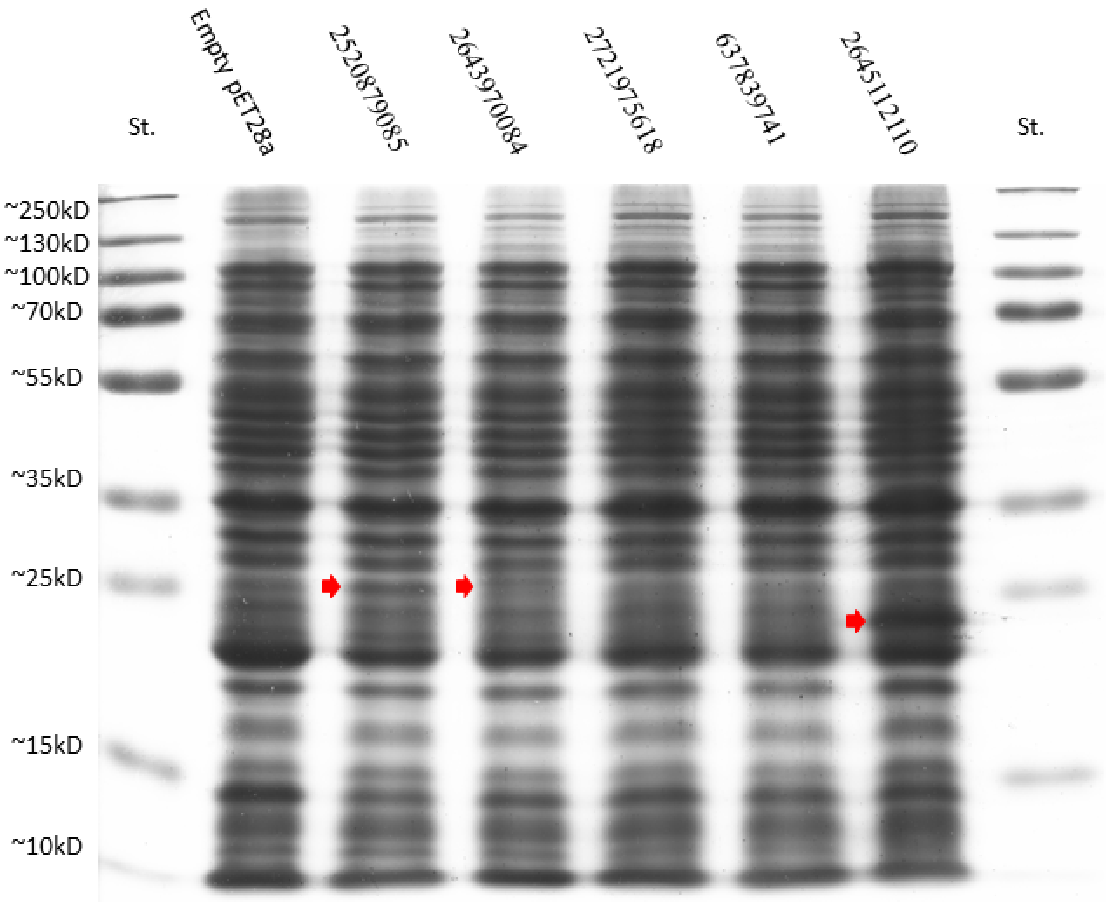

Supplementary Figure 22. Protein expression of failed EATs. Coomassie brilliant blue stained SDS-PAGE of whole cell lysates. In wells (from left to right): St.: PageRuller™ plus protein standards (ThermoFisher scientific) with approximate molecular weights on the left. Empty pET28a: BL21 (DE3) pLysS *E. coli* strain carrying empty pET28a lysate. 3-7: IMG gene ID of genes cloned into pET28a vectors in BL21 (DE3) pLysS *E. coli* strain. Red Arrows show unique bands in IPTG induced cells on the approximate molecular weights predicted for translation products of genes. This experiment was performed once.

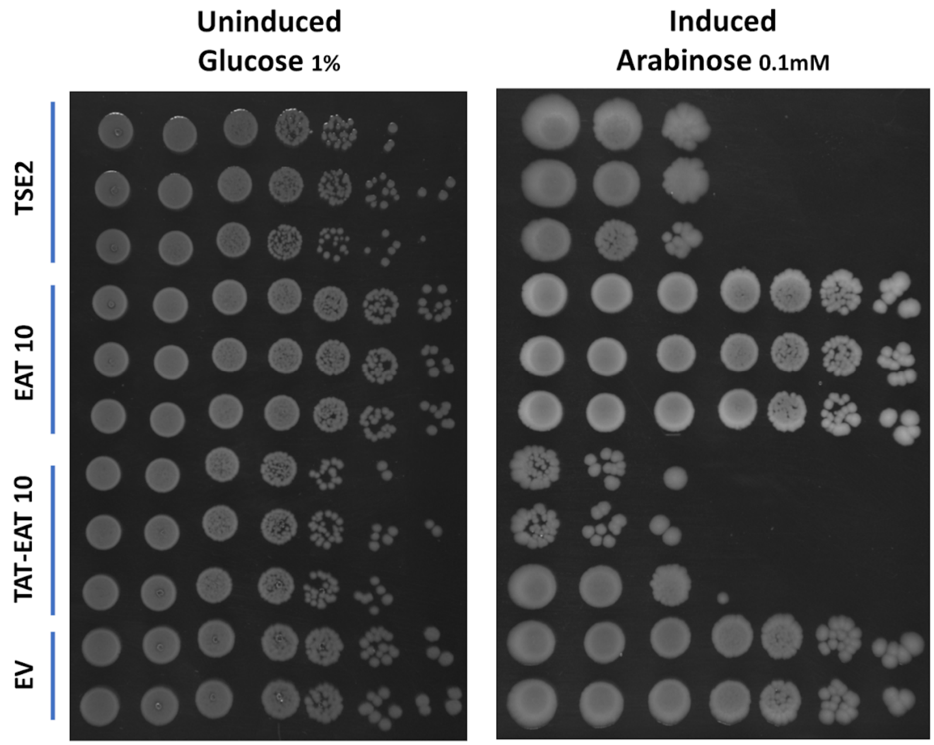

Supplementary Figure 23. Addition of TAT sequence upstream to EAT10 increases into toxicity towards *E. coli* cells. All genes were expressed in pBAD24 (in comparison to expression in pET29 in Figure 4). TSE2 represents a positive control (known toxin), EV: empty vector. EAT10 is cytoplasmically localized; TAT-EAT10 has an N-terminal TAT sequence for periplasmic localization. Each condition has three biological replicates. Overnight cultures of each strain were grown, normalized for uniform OD, and serially diluted and plated in drops on LB with the appropriate antibiotics. In repression conditions (1% Glucose), all strains grow comparably; upon induction (0.1 mM Arabinose), only the periplasmically-localized EAT10 is toxic to *E. coli*.

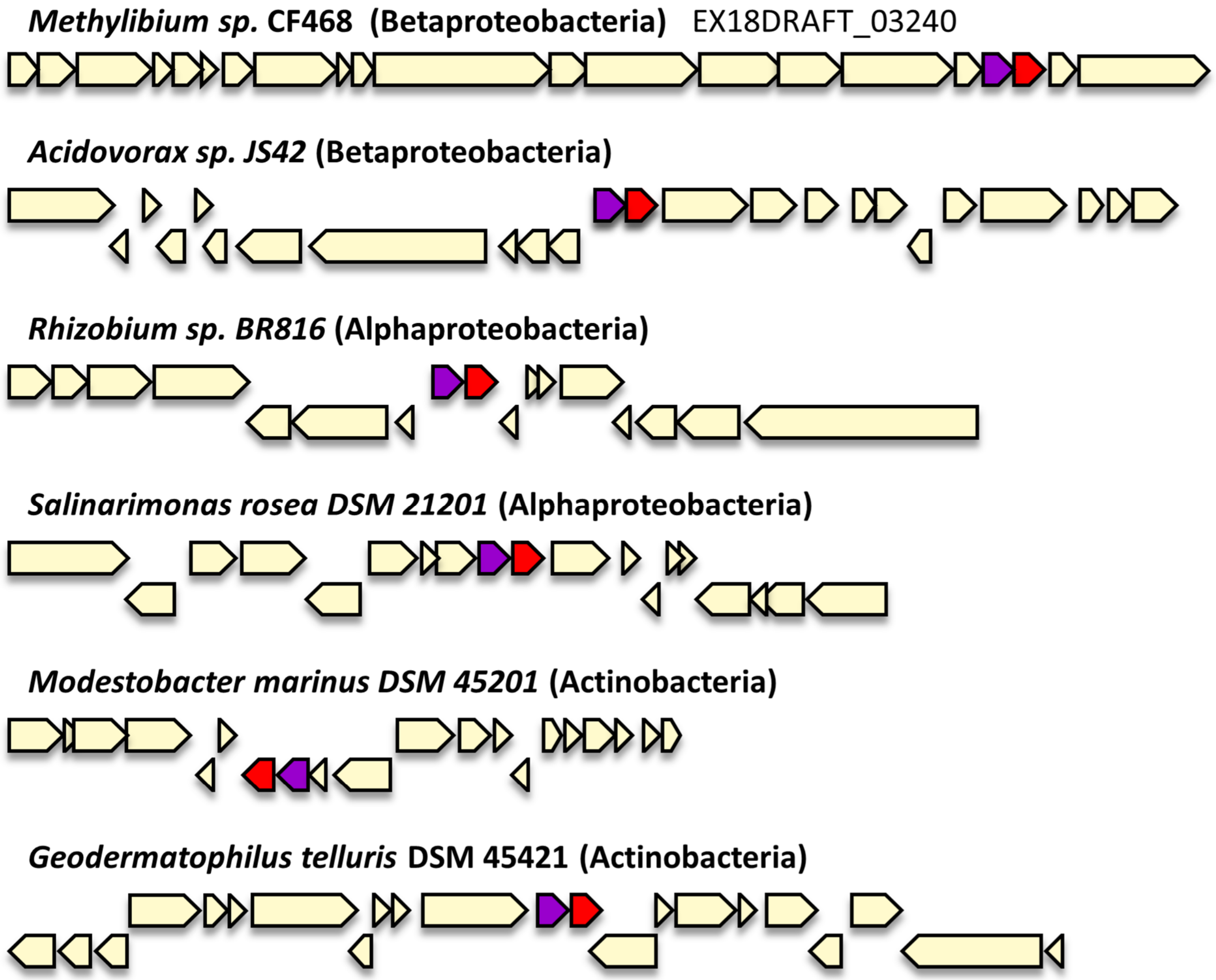

Supplementary Figure 24. RES-Xre Gene pair synteny. A gene pair containing the gene for EAT5 is conserved through various strains from Proteobacteria and Actinobacteria. All occurrences except for the top one are outside eCIS genomic context (IMG locus tag: Ajs_1583; RhiBR816DRAFT_0322 ;Swit_5317; Ga0056099_04803 ;Ga0056090_4110). Based on this synteny, we predicted that the purple gene next to EAT5 serves as an antitoxin.

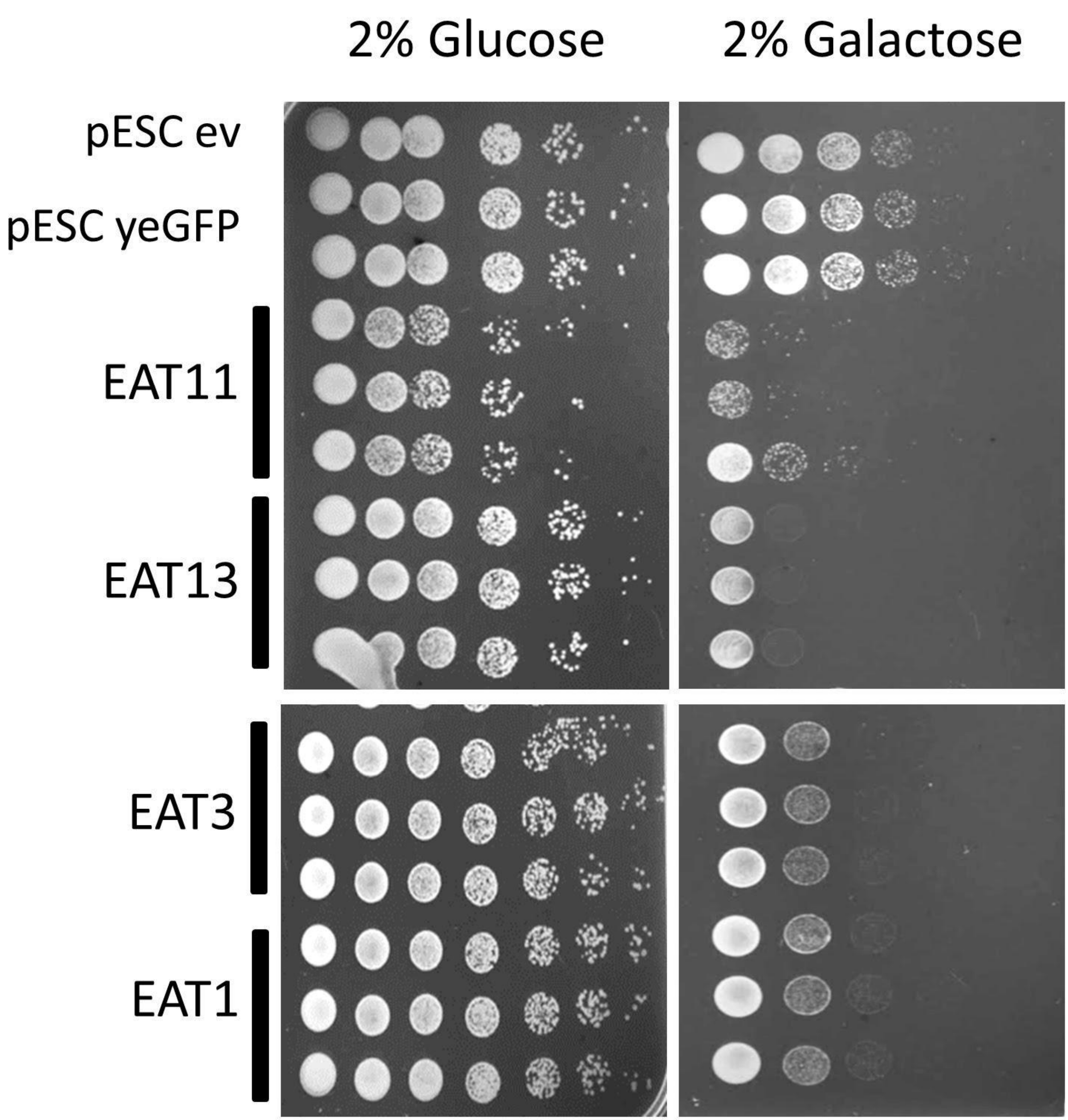

Supplementary Figure 25. Biological replicates of EATs killing *Saccharomyces cerevisiae*. EATs were cloned into pESC -leu Galactose inducible plasmids that were then transformed into *Saccharomyces cerevisiae* BY4742 strain. Overnight cultures of the strains harboring the vectors of interest were grown in SD -leu media. The cultures OD was normalized and then washed once with water and split into two: one part was grown overnight in repressive conditions (SD -leu + 2% glucose) and the other part was grown in inductive conditions (SD -leu +

2% galactose). Dilutions were spotted on SD -leu plates containing glucose or galactose and the plates were incubated two nights at 30C. Negative controls: empty vector (ev) and non-toxin (yeGFP gene).

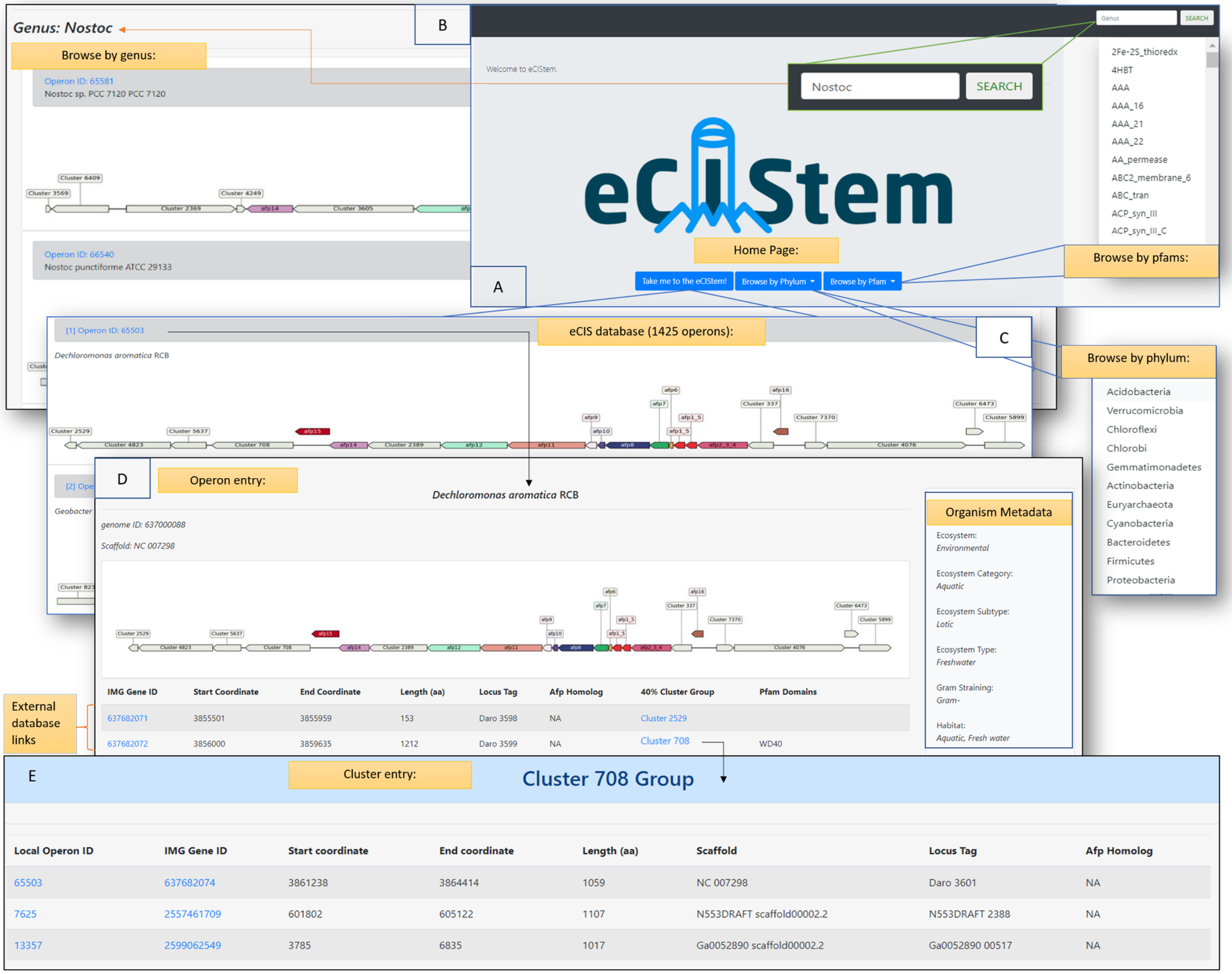

Supplementary Figure 26. eCIStem database provides information on 1425 eCIS operons and their encoding genomes. Arrows represent links from page to page. A. eCIStem homepage B. A list of operons filtered by “Nostoc” genus name. C. A view of eCIS gene operons. D. An operon entry with information of each gene in the operon with operon, gene, protein, and protein domain information. E. Examples of eCIS protein cluster (clustered by 40% amino acids sequence identity).

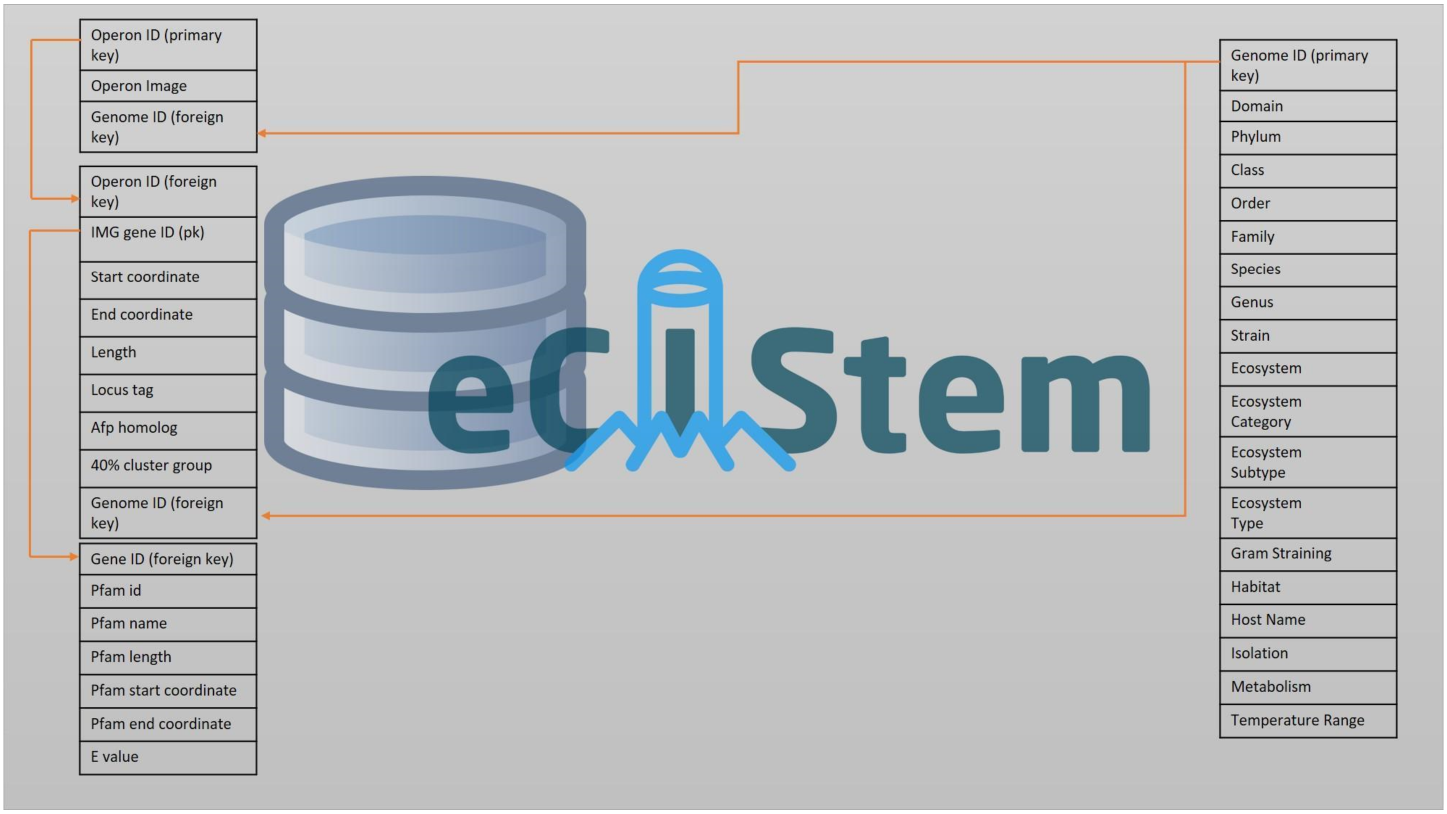

Supplementary Figure 27. eCIStem database scheme. eCIStem database is built on sqlite database engine. SQLite it is a relational database management system embedded in django framework. It contains 4 tables: Operons Table with primary key (i.e. unique) field named "Operon ID", Genes Table with primary key field named "IMG gene ID", Genome Table with primary key named "Genome ID" and PFAMs Table with Pfam ID as a unique primary key field. The tables are linked by "one to many" relationships that are pointed by orange arrows. This relationship was made for making efficient queries implementation. Sqlite database stored as a single file on our host server.

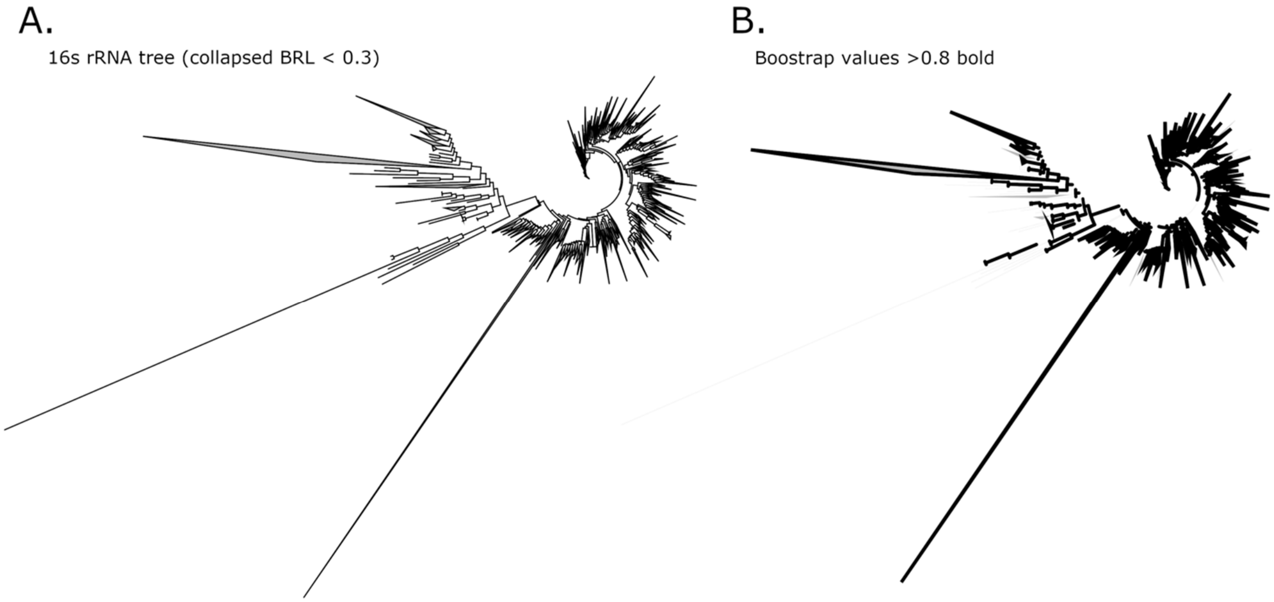

Supplementary Figure 28. 16s Tree Used For Scoary. 16s Maximum likelihood tree used for Scoary analysis (Methods) is shown in A. with collapsed branches with branch length (BRL) that are closer than 0.3 units to one another. B. Branches of the same tree with >0.8 values for bootstrap are shown in bold.

Association	domain type	domain ID	domain name	summary
eCIS	pfam	PF00004	AAA	ATPase family associated with various cellular activities (AAA)
eCIS	pfam	PF04865	Baseplate_J	Baseplate J-like protein
eCIS	pfam	PF04965	GPW_gp25	Gene 25-like lysozyme
eCIS	pfam	PF04984	Phage_sheath_1	Phage tail sheath protein subtilisin-like domain
eCIS	pfam	PF05488	PAAR_motif	PAAR motif
eCIS	pfam	PF05954	Phage_GPD	Phage late control gene D protein (GPD)
eCIS	pfam	PF06841	Phage_T4_gp19	T4-like virus tail tube protein gp19
eCIS	pfam	PF14065	DUF4255	Protein of unknown function (DUF4255)
phage	pfam	PF04860	Phage_portal	Phage portal protein
phage	pfam	PF03237	Terminase_6	Terminase-like family
phage	pfam	PF17289	Terminase_6C	Terminase RNaseH-like domain
phage	pfam	PF05929	Phage_GPO	Phage capsid scaffolding protein (GPO) serine peptidase
phage	pfam	PF05125	Phage_cap_P2	Phage major capsid protein, P2 family
phage	pfam	PF05944	Phage_term_smal	Phage small terminase subunit
phage	pfam	PF05926	Phage_GPL	Phage head completion protein (GPL)
phage	pfam	PF04550	Phage_holin_3_2	Phage holin family 2
phage	pfam	PF05840	Phage_GPA	Bacteriophage replication gene A protein (GPA)
phage	pfam	PF04606	Ogr_Delta	Ogr/Delta-like zinc finger
phage	pfam	PF05065	Phage_capsid	Phage capsid family
phage	pfam	PF07068	Gp23	Major capsid protein Gp23
phage	pfam	PF16855	Soc	Small outer capsid protein
phage	pfam	PF06810	Phage_GP20	Phage minor structural protein GP20
phage	pfam	PF03864	Phage_cap_E	Phage major capsid protein E
phage	pfam	PF02924	HDPD	Bacteriophage lambda head decoration protein D
phage	pfam	PF10124	Mu-like_gpT	Mu-like prophage major head subunit gpT
T6SS	COG	COG0542	ClpA	ATP-dependent Clp protease ATP-binding subunit ClpA [Posttranslational modification, protein turnover, chaperones]
T6SS	COG	COG3521	COG3521	Predicted component of the type VI protein secretion system [Intracellular trafficking, secretion, and vesicular transport]
T6SS	COG	COG3523	IcmF	Type VI protein secretion system component VasK [Intracellular trafficking, secretion, and vesicular transport]

Supplementary Table 1. Domains used to search IMG database. pfam/COG IDs of genes used to search for eCIS operons, and domains used to exclude phage and T6SS operons.

No.	EAT No.	Species encoding putative toxin	IMG Gene ID	Organism tested	Toxicity to Yeast (y / n)	Toxicity to *E. coli* (y / n)
1	EAT 4	Mucilaginibacter sp. OK098	2609591113	*Yeast / E. coli*	n	y
2		Flavobacterium sp. F52	2520879085	*E. coli*		n
3	EAT 5	Methylibium sp. CF468	2587734256	*E. coli*		y
4	EAT 3	Dickeya zeae Ech1591	644851057	*Yeast / E. coli*	y	y
5	EAT 2	Flavobacterium sp. FV08	2616189889	*Yeast / E. coli*	n	y
6		Rhizobacter sp. Root16D2	2643970084	*E. coli*		n
7	EAT 1	Rhizobacter sp. Root29	2644141250	*Yeast / E. coli*	y	y
8	EAT 12	Ruminiclostridium cellulolyticum H10	643608442	*E. coli*		y
9		Bacteroidetes bacterium GWC2_46_850	2721975618	*Yeast / E. coli*	n	n
10	EAT 11	Amycolatopsis nigrescens CSC17Ta-90, DSM 44992	2515141031	*Yeast / E. coli*	y	y
11		Burkholderia thailandensis E264, ATCC 700388	637839741	*E. coli*		y
12		Porphyromonadaceae bacterium COT-184	2629491311	*E. coli*		y
13	EAT 9	Moorea producens PAL 15AUG08-1	2631153083	*Yeast / E. coli*	n	y
14		Chryseobacterium sp. Leaf404	2645112110	*Yeast / E. coli*	n	y
15	EAT 8	Paenibacillus jilunlii CGMCC 1.10239	2668039404	*Yeast / E. coli*	n	y
16	EAT 6	Parafilimonas terrae DSM 28286	2695001213	*Yeast / E. coli*	n	y
17	EAT 10	Pseudoalteromonas luteoviolacea S4054249	2720476818	*E. coli*		y
18	EAT 7	Janthinobacterium sp. 551a	2602024580	*E. coli*		y
19	EAT 13	Candidatus Udaeobacter copiosus	2653242303	*Yeast*	y	
20		Tenacibaculum sp. Mar_2010_89	2636399931	*Yeast*	n	
21		Caulobacter sp. Root656	2643927987	*Yeast*	n	

Supplementary Table 2. All genes tested for toxicity. Genes that were heterologously expressed in *E. coli* and/or *Saccharomyces cerevisiae* and whether they were toxic (y) or non-toxic (n) are listed.
