# Supplementary material for: The extracellular contractile injection system is enriched in environmental microbes and associates with numerous toxins
Source: Nat Commun. 2021 Jun 18;12:3743. doi: 10.1038/s41467-021-23777-7 (PMC8213781; doi:10.1038/s41467-021-23777-7)
Supplement: Supplementary file 3 — Description of Additional Supplementary Files [file 41467_2021_23777_MOESM3_ESM.pdf]

Description of Additional Supplementary Files

File Name: Supplementary Data 1
Description: **64,756 Public IMG genomes.** All genomes queries for this study, with Phylum, Genus, Species, Strain and accession ID. Those containing eCIS are marked.

File Name: Supplementary Data 2
Description: **eCIS operons.** Information about the 1425 eCIS operons we identified and their encoding organisms.

File Name: Supplementary Data 3
Description: **Genes included in eCIS operons**.

File Name: Supplementary Data 4
Description: **Alignment of Afp11 and Afp8 concatenation used to build maximum likelihood tree.** Alignment in Fasta format.

File Name: Supplementary Data 5
Description: **eCIS predicted to be carried on plasmids.** We ran Deeplasmid tool on all contigs/scaffolds carrying eCIS operon and annotated plasmids based on Deeplasmid score >0.7. Deeplasmid is a deep learning based classifier that identifies plasmids based on presence of plasmidic genes and other features such as coding density, and GC content.

File Name: Supplementary Data 6
Description: **Correlation between eCIS and genera.** For each genus in our genomic database we performed a Fisher exact test by counting the number of sequenced genomes within the genus encoding eCIS, those that lack eCIS, genomes from all other genera that encode eCIS, and enomes from all other genera that lack eCIS.

File Name: Supplementary Data 7
Description: **Genomes containing multiple eCIS operons**.

File Name: Supplementary Data 8
Description: **eCIS correlation with microbial ecological and physiological features.** All data were downloaded from IMG database.

File Name: Supplementary Data 9
Description: **BLAST results of Apf13 proteins against proteins from viruses and phages.** The best hits are presented. Tabs include also the Afp13 genes that were queried and the taxon ids of viruses and phages used as targets.

File Name: Supplementary Data 10
Description: **Pfam enrichment in eCIS operons.** Results of Fisher exact test for enrichment of the different Pfam domains within proteins encoded by eCIS operons.

File Name: Supplementary Data 11
Description: **eCIS-associated genes with toxin domains.**

File Name: Supplementary Data 12
Description: **eCIS Accessory Gene and Core Gene Clusters.** The "eCIS Accessory Genes Cluster" tab shows all accessory proteins (i.e. non-AFP homologs), clustered by at least 40% identity over at least 80% of each of their lengths. Cluster ID = a unique nominal identifier for each 40% identity group. Blank entries in Conserved Domain columns represent no hit from the NCBI CDD database. Widespreadness refers to each cluster, and how many of the indicated taxa are represented in each cluster. Operon ID eCIStem Database is a unique identifier for the eCIStem database (see full text for more information). The "eCIS Core Gene Clusters" tab shows clusters of core genes (i.e. AFP homologs). Each cluster is a unique nominal identifier for genes with at least 40% ID over at least 80% of clusters.
